# Global prevalence and sex differences in rheumatic heart disease: a systematic review and updated meta-analysis

**DOI:** 10.3389/fcvm.2025.1615158

**Published:** 2025-11-06

**Authors:** Antonio Mutarelli, Alexandre N. Pantaleao, Pedro H. C. Melo, Wilson Nogueira, Alleh Nogueira, Nicole Felix, Giuliano Generoso, Rhanderson Cardoso, Adrien Lupieri, Elena Aikawa, Robert A. Levine, Maria C. P. Nunes

**Affiliations:** 1 School of Medicine, Federal University of Minas Gerais, Belo Horizonte, Brazil; 2 Cardiac Ultrasound Lab, Massachusetts General Hospital, Harvard Medical School, Boston, MA, United States; 3 The Cardiovascular Research Foundation, New York, NY, United States; 4 School of Medicine, State University of Piauí, Teresina, Brazil; 5 Medical and Public Health School of Bahia, Salvador, Brazil; 6 School of Medicine, Federal University of Campina Grande, Campina Grande, Brazil; 7 Division of Cardiology, Sirio-Libanes Hospital, São Paulo, Brazil; 8 Center for Clinical and Epidemiological Research, Medical School, São Paulo University, São Paulo, Brazil; 9 Heart and Vascular Center, Brigham and Women’s Hospital, Harvard Medical School, Boston, MA, United States; 10 The Center for Excellence in Vascular Biology, Cardiovascular Medicine, Brigham and Women’s Hospital, Harvard Medical School, Boston, MA, United States; 11 The Center of Interdisciplinary Cardiovascular Sciences, Cardiovascular Medicine, Brigham and Women’s Hospital, Harvard Medical School, Boston, MA, United States

**Keywords:** rheumatic heart disease, prevalence, sex disparity, meta-analysis, latent RHD

## Abstract

**Background:**

Rheumatic heart disease (RHD), a sequela of acute rheumatic fever (ARF), remains as the leading cause of acquired cardiac disease in children, posing a significant burden to health systems, especially in low-to-middle-income countries. While ARF shows equal prevalence among sexes in children, clinically manifest RHD in adulthood is strikingly more prevalent in females, with at least a 2:1 ratio. We conducted a meta-analysis to evaluate the global prevalence of RHD and sex disparities alongside risk factors.

**Methods:**

PubMed, Embase, Cochrane, and Lilacs were searched for cross-sectional studies on RHD prevalence in individuals aged 5–20, evaluated through echocardiogram-based screening in endemic areas. Studies relying on auscultation were excluded. RHD was defined as borderline/definite by 2012 WHF criteria or possible/probable/definite by WHO criteria.

**Results:**

Fifty-eight studies with 215,552 subjects were included. Echo-detected RHD prevalence was 24/1,000 (95%-CI: 20-30) globally. Subgroup analyses showed consistently lower RHD prevalence in males (RR: 0.70; 95%-CI: 0.61–0.80; *p* < 0.01). Definite RHD prevalence was 9/1,000 (95%-CI: 7–12), with lower rates among males (RR: 0.71; 95%-CI: 0.59–0.86; *p* < 0.01). Children in private schools (RR: 0.68; 95%-CI: 0.48–0.97; *p* = 0.03), medium-high-income families (RR: 0.57; 95%-CI: 0.41–0.81; *p* < 0.01), and urban areas (RR: 0.49; 95%-CI: 0.26–0.93; *p* = 0.03) exhibited reduced RHD prevalence.

**Conclusion:**

This meta-analysis highlights early gender disparities in RHD, with female predominance preceding established valve lesions. Prevalence remains higher in rural areas, public schools, and low-income families, with global prevalence in endemic regions at 24/1,000.

**Systematic Review Registration:**

https://www.crd.york.ac.uk/PROSPERO/view/CRD42023491941, PROSPERO CRD42023491941.

## Introduction

Rheumatic heart disease (RHD) disproportionately affects developing countries and remains the predominant acquired heart disease among the young in these regions (1). According to the most recent Global Burden of Disease estimates, RHD prevalence has decreased in high-income countries (2); however, it has increased in low-to-middle-income countries, affecting nowadays over 40 million patients worldwide (2). RHD also accounts for more than 300,000 annual deaths and nine million disability-adjusted life years lost (3).

RHD development involves complex interactions among host susceptibility factors, with sex predisposition playing a crucial role (4, 5). The prevalence ratio of acute rheumatic fever (ARF) between males and females is unclear, but most evidence supports equal prevalence (4, 6, 7). Despite that, the risk of heart valve damage from RHD significantly rises in adult females, especially between the ages of 25 and 45 (8, 9). Chronic RHD has a clear female predominance, with ratios ranging from 2:1 to 4:1 in different studies (9, 10). Nonetheless, the precise age when these sex differences become evident is still a gap in the literature. This epidemiological data is crucial to stimulate further research in sex predisposition in RHD, increasing our understanding of RHD pathophysiology and potentially improving disease management through new therapeutic targets.

Early identification of RHD, prior to established valvular dysfunction, and the prompt initiation of penicillin prophylaxis may prevent disease progression and reduce RHD burden worldwide (1). Moreover, a double-blind placebo-controlled trial indicated that regular use of penicillin G benzathine every four weeks can significantly reduce the risk of progression of echocardiographically detected RHD (11). RHD burden tends to be highest in resource-limited countries owing to social disparities and lack of access to adequate diagnostic tools and therapies. Echocardiographic screening for RHD allows early detection, supporting timely decision-making for initiating antibiotic prophylaxis. (12, 13).

Our study aims to provide current insights into the prevalence of RHD across continents, ages, and risk factors, with a particular focus on sex disparities. Aligned with research priorities set by both the American Heart Association and World Heart Federation (WHF), we conducted a systematic review and updated meta-analysis of the prevalence, severity, and variations of RHD across clinical and socioeconomic subgroups (1, 14). This is a summary of the literature prior to the application of 2023 WHF diagnostic criteria for RHD screening (15).

## Methods

This systematic review and meta-analysis were conducted in line with Cochrane recommendations and Preferred Reporting Items for Systematic Reviews and Meta-Analysis (PRISMA) statement guidelines (16, 17). Accordingly, it was prospectively registered with the International Prospective Register of Systematic Reviews (PROSPERO number CRD42023491941).

### Eligibility criteria

We restricted inclusion in this meta-analysis to: (i) population-based studies published in English; (ii) that analyzed individuals aged 5–20 years; (iii) from areas with a high burden of RHD; (iv) with a sample size of at least 500 subjects; and (v) reported the prevalence of echocardiogram-assessed latent RHD. We excluded conference abstracts, studies with overlapping populations, retrospective studies, prospective studies not primarily focused on screening of latent RHD, or studies including specific subgroups such as individuals with symptoms of RHD, abnormal cardiac auscultation, or family history of RHD. Studies published in languages other than English were excluded.

RHD was identified through echocardiographic screening in asymptomatic individuals (18). We considered RHD as borderline or definite by the 2012 WHF diagnostic criteria and possible, probable, or definite by the World Health Organization (WHO) diagnostic criteria (WHO criteria were defined by an expert panel under WHO and National Institutes of Health supervision in 2005) (19). Additional information on the diagnostic criteria is available in the supplement.

### Search strategy and data extraction

We systematically searched four databases (PubMed, Excerpta Medica Database [Embase], Latin American and Caribbean Center on Health Sciences Information [LILACS], and the Cochrane Central Register of Controlled Trials) from inception to November 2024 using combined subject headings including rheumatic, heart, cardiopathy, valvular, prevalence, screening, surveillance, epidemiology, child, teenager, adolescent, and school. The full search string applied to each database is available in Supplementary Table S1.

To avoid missing data, we proactively requested pertinent information from the authors of the selected articles. Two investigators (AM and ANP) independently assessed search results according to predefined criteria to identify eligible studies. Four investigators (AM, ANP, PHCM, and WN) worked in pairs to extract key study characteristics and endpoints. In both instances, any disagreements were resolved through consensus.

### Endpoint definition and subgroups

The study endpoint was the echocardiographic prevalence of RHD in endemic areas. Secondary endpoints encompassed prevalence (i) by continent; (ii) of borderline RHD and definite RHD by 2012 WHF criteria; (iii) by age; (iv) by disease severity; and (v) of the echocardiographic findings.

We also performed the first meta-analysis directly comparing RHD prevalence between different subgroups: (i) males and females; (ii) population from rural and urban areas; (iii) children from private and public schools; and (iv) individuals from low- and medium-to-high income families.

### Quality assessment

Two investigators (AM and ANP) independently evaluated the quality of each included article using the risk of bias assessment tool for prevalence studies established by Hoy et al (20). Any discrepancies in the quality assessment were resolved through consensus. The assessment by Hoy et al. comprises ten items categorized into two groups: external validity and internal validity (20).

Each item is given a score of 1 (indicating yes) or 0 (indicating no). The aggregate scores yield an overall quality assessment that categorizes the study as having either a low, moderate, or high risk of bias. A score of 7 or higher indicates a study with a low risk of bias, while 4–6 signifies moderate risk and 3 or lower indicates high risk.

We investigated the potential for small study effects that might be associated with publication bias by closely evaluating funnel plots and assessing the distribution of point estimates against their standard errors. Furthermore, when the number of studies exceeded ten, we conducted Egger's regression analysis as a formal statistical test to detect funnel plot asymmetry (21).

### Statistical analysis

To accommodate the anticipated between-study heterogeneity stemming from differences in study populations, assessments, and settings, we calculated binary event prevalence with 95% confidence intervals (CI) using random-effects generalized linear mixed models (GLMM). We applied the Mantel-Hazel random-effects model to pool prevalence ratios (RR) with 95% CI for secondary analyses directly comparing the incidence of events in specific subgroups. *P*-values less than 0.05 were deemed statistically significant.

Estimates from individual studies were pooled using generalized inverse variance weighting. We examined the influence of continuous and categorical covariates on the prevalence or RR of binary events through meta-regressions. The impact of between-study heterogeneity on the estimates was assessed using Cochran's *Q* test and the *I^2^* statistic. In accordance with Cochrane guidelines, we regarded a *p*-value of less than 0.10 or an *I^2^* greater than 40% as indicating substantial heterogeneity affecting the estimates (17).

As part of sensitivity analyses, we conducted meta-regressions and leave-one-out analyses to identify sources of heterogeneity and potential effect modifications in the estimated outcomes. All statistical analyses were independently performed by two authors (AM and AN) using R version 4.3.0 (R Foundation for Statistical Computing, Vienna, Austria) (22).

## Results

### Study selection and characteristics

**Figure 1:**
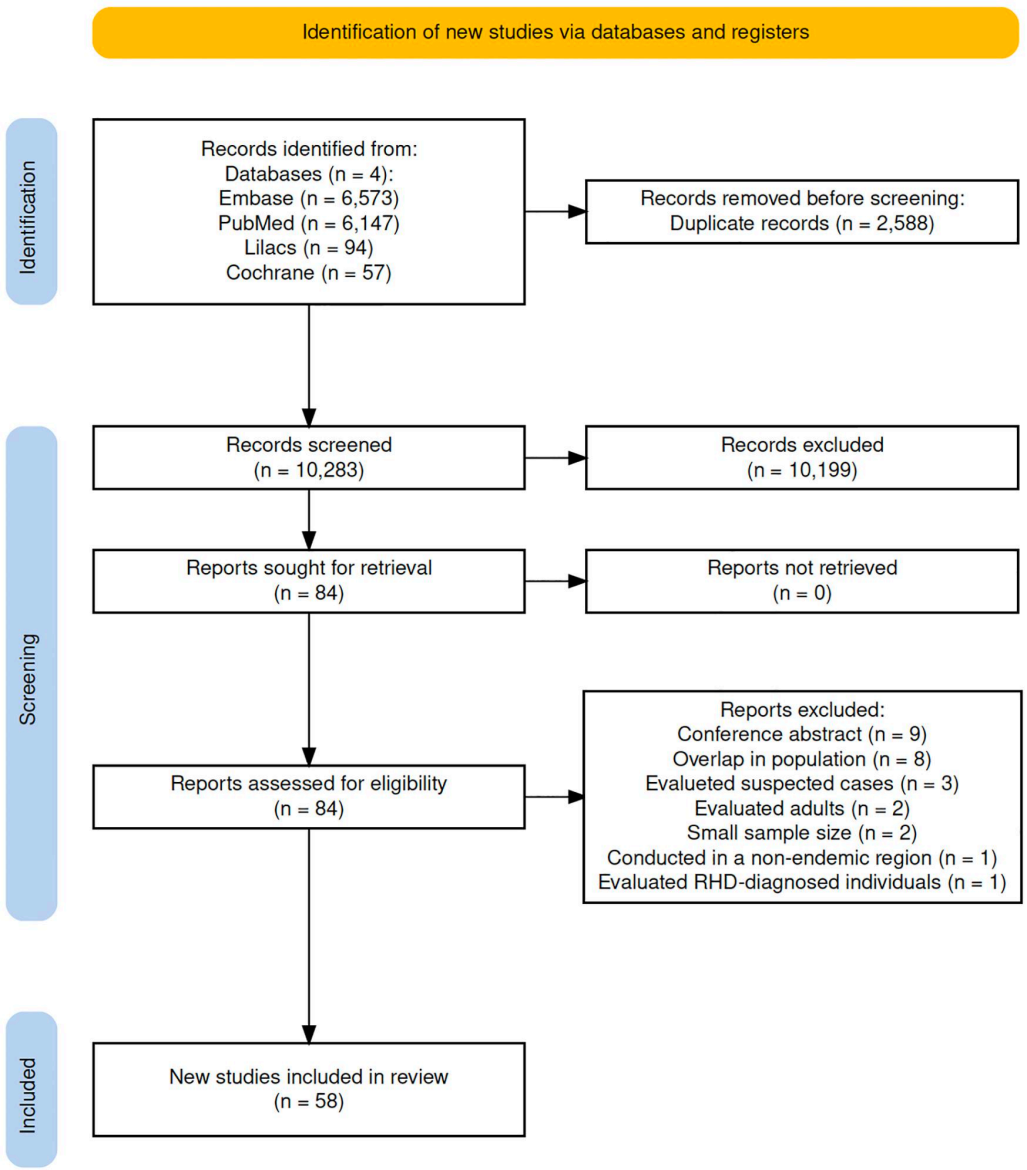
Flow diagram of study selection.

The initial search yielded 12,871 articles and conference abstracts. Following the removal of 2,588 duplicates, we screened 10,283 articles, of which 10,199 were excluded based on title and abstract screening. Subsequently, we evaluated 84 full-text manuscripts for eligibility, ultimately including 58 that met our criteria, and provided data on latent RHD prevalence (Figure 1) (12, 13, 23–78). This analysis encompassed a total of 215,552 screened children and adolescents. The diagnostic criteria used included WHF criteria in 46 studies, WHO criteria in eight, and other criteria (criteria based on the WHF with only one echocardiographic view) in four.

Additional information regarding the excluded studies is available in the supplement (Supplementary Table S2). Seven studies investigated the difference in RHD prevalence among children enrolled in public vs. private schools. Ten studies directly compared RHD prevalence between rural and urban populations, while 38 studies furnished data on RHD prevalence among both male and female participants. The supplement depicts the methodological and baseline clinical characteristics of the included studies (Supplementary Table S3).

Briefly, participant recruitment spanned from 2001 to 2022, with individual study sample sizes varying between 522 and 16,294. Most of the studies [48 (83%)] were conducted within school settings. Africa was the most frequently represented region in terms of study count, with 27 studies, and in terms of the highest number of children and adolescents screened, totaling 73,304 (34%). In contrast, Latin America was least represented, featuring three studies and 16,221 (7.5%) screened children and adolescents.

### Prevalence of RHD

**Figure 2:**
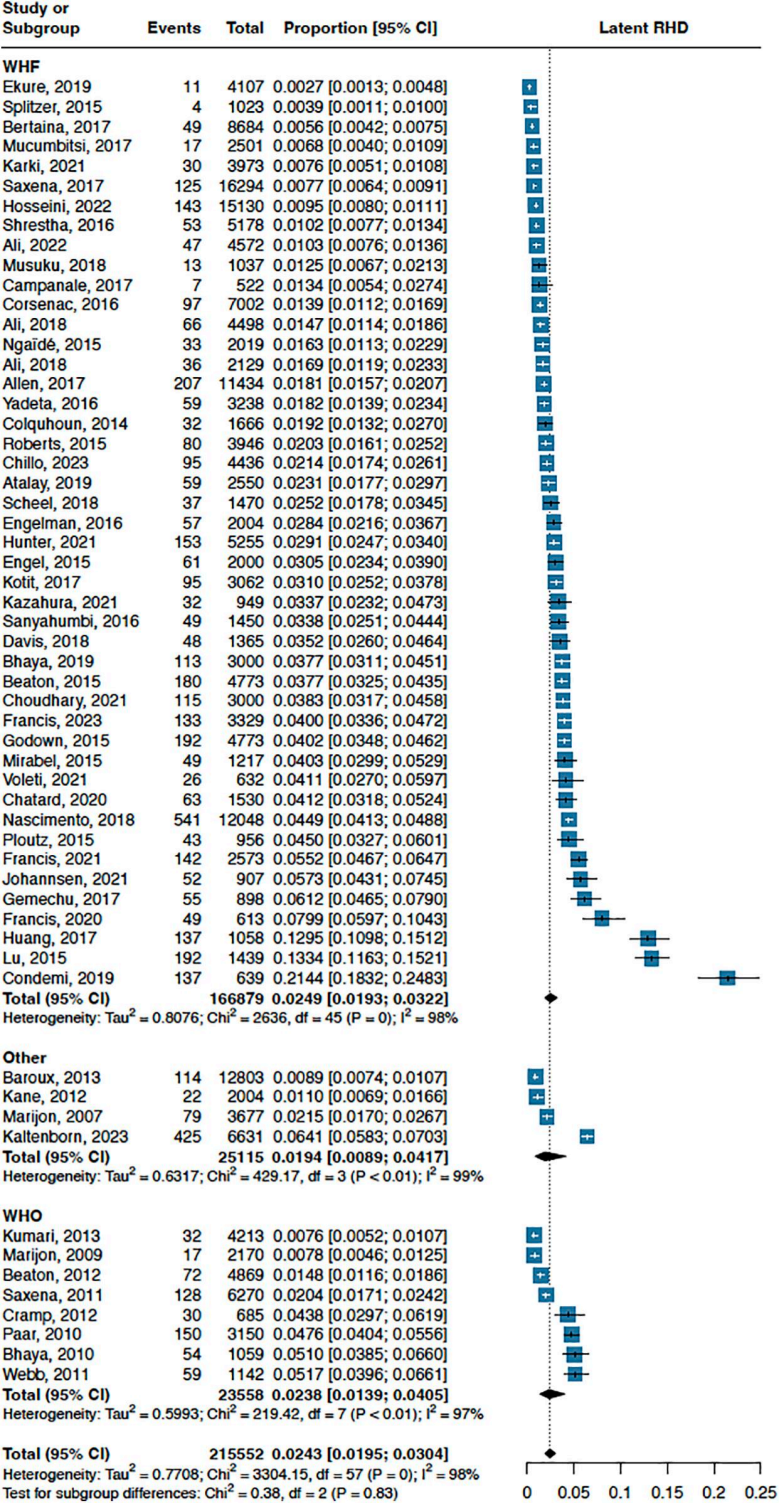
Prevalence of latent RHD with different diagnostic criteria (WHF criteria and wHO criteria).

**Figure 3:**
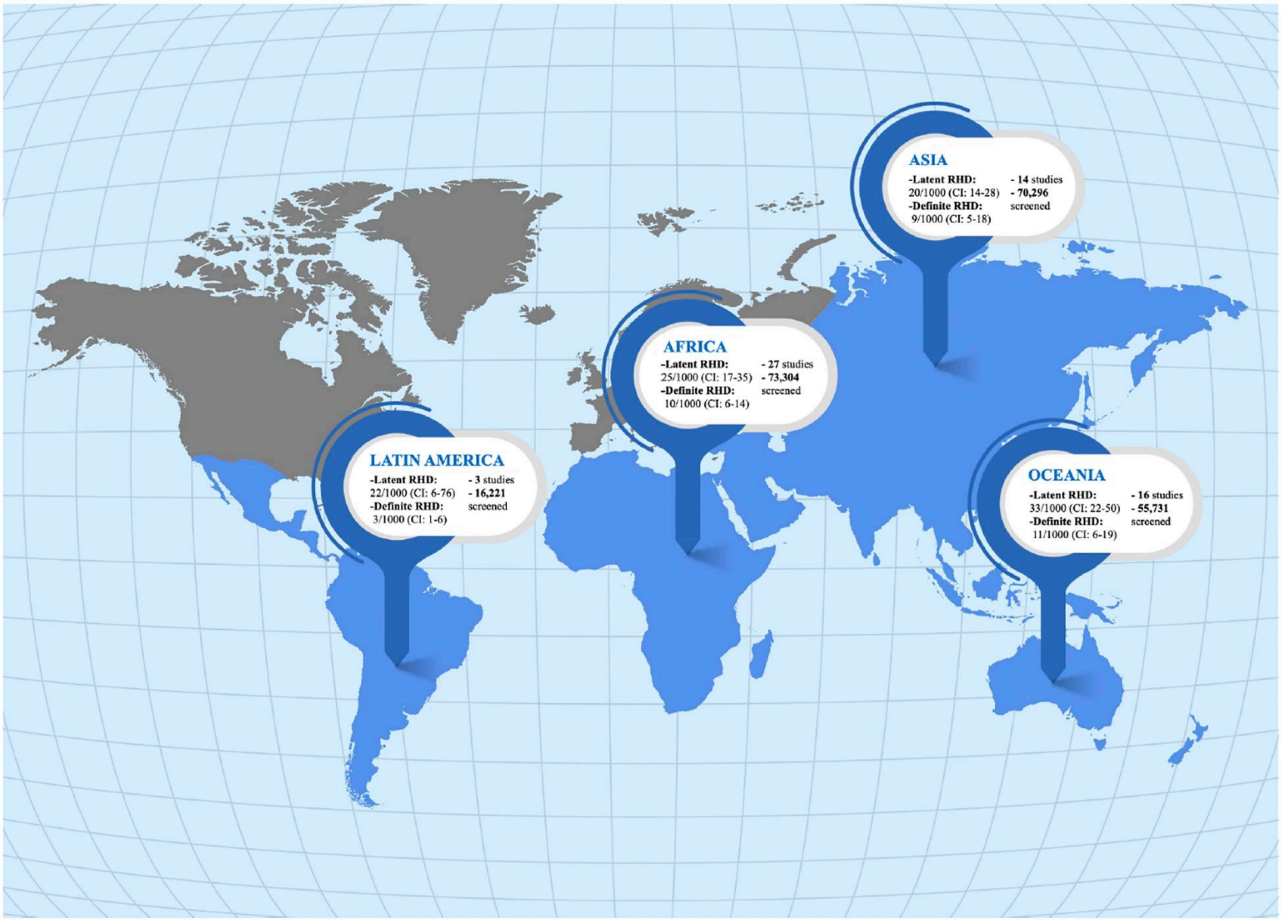
Consistent prevalence of latent RHD globally across endemic areas.

The overall prevalence of echo-detected RHD was 24 per 1000 individuals (95% CI 20–30; I^2^ = 98%; Figure 2), with similar findings across the continents (Figure 3). When evaluating prevalence using the criteria outlined by both the WHO and the WHF, the definite RHD prevalence rate was 9 per 1,000 individuals (95% CI 7–12; I^2^ = 97%; Supplementary Figure S1).

**Figure 4:**
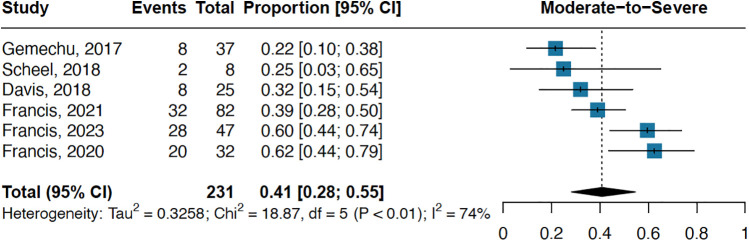
Prevalence of 41% of moderate-to-severe definite RHD.

Six studies investigated the prevalence and severity of valvular lesions at the time of diagnosis in definite RHD, revealing moderate-to-severe lesions in 41% (95% CI 28–55; I^2^ = 74%; Figure 4). In a more detailed analysis focusing on the 2012 WHF criteria, we scrutinized each diagnostic criterion for both definite RHD (19). The prevalence of criteria A, B, C, and D for definite RHD is illustrated in the supplement (Supplementary Figure S7), with criterion A (mitral regurgitation accompanied by two or more morphological features of RHD) demonstrating the highest prevalence, at 82% (95% CI 75–87; I^2^ = 50%; Supplement Figure S7). Complete echocardiographic diagnostic criteria for RHD, as specified by the WHF, are illustrated in the supplement (Supplementary Figure S7).

**Figure 5:**
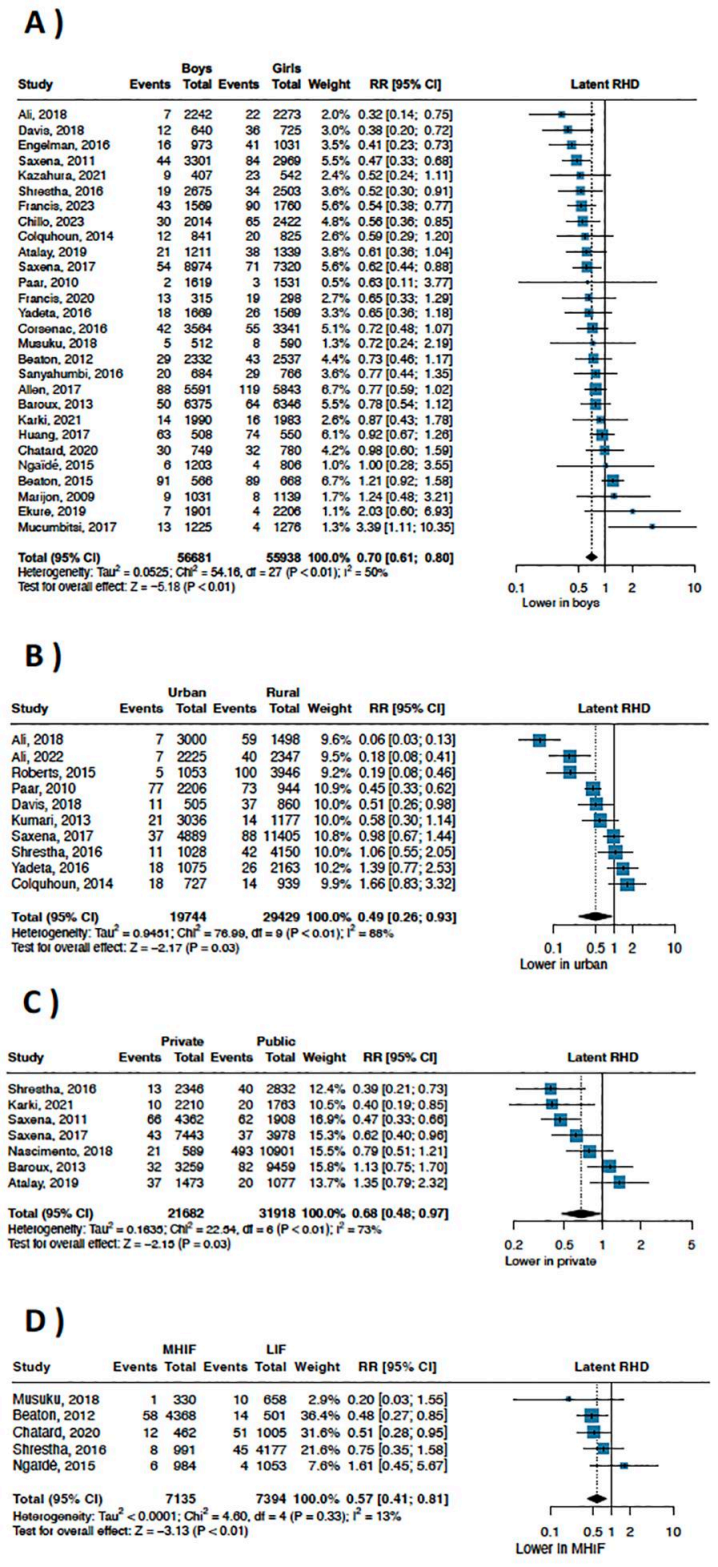
There was a significantly lower prevalence of RHD in males vs. females **(A)** urban vs. rural areas **(B)**; private vs. public schools **(C)**; medium-high vs. low-income families **(D).**

Among the 28 studies that reported latent RHD prevalence data across sex, there was a lower prevalence in males (RR 0.70; 95% CI 0.61–0.70; *p* < 0.01; I^2^ = 50%; Figure 5A). Within this set of 28 studies, 15 provided figures for definite RHD, corroborating a lower prevalence among males (RR 0.71; 95% CI 0.59–0.86; *p* < 0.01; I^2^ = 0%; Supplementary Figure S5).

Ten studies directly compared RHD prevalence between rural and urban populations, uncovering a lower urban prevalence (RR 0.49; 95% CI 0.26–0.93; *p* = 0·03; I^2^ = 88%; Figure 5B). Seven studies assessed RHD prevalence among children attending public and private schools, demonstrating a lower prevalence among children in private schools (RR 0.68; 95% CI 0.48–0.97; *p* = 0.03; I^2^ = 73%; Figure 5C). Five studies compared RHD prevalence between low- and medium-to-high income families, with a lower prevalence in medium-to-high income families (RR 0.57; 95% CI 0.41–0.81; *p* < 0.01; I^2^ = 13%; Figure 5D).

Oceania had the highest prevalence rate among all continents, 33 per 1,000 individuals (95% CI 22–50; I^2^ = 98%; Figure 3 and Supplementary Figure S4), followed by Africa (25 per 1,000, 95% CI 17-35; I^2^ = 98%), Latin America (22 per 1,000, 95% CI 6-76; I^2^ = 92%), and Asia (20 per 1,000, 95% CI 14-28; I^2^ = 98%).

When analyzing the prevalence of echo-detected RHD by WHO regions, the Western Pacific revealed the highest rate, with a prevalence of 31 per 1,000 individuals (95% CI 21–47; I^2^ = 98%; Supplementary Figure S5). African region had a prevalence of 24 per 1,000 (95% CI 17–34; I^2^ = 98%), while the Americas presented a prevalence rate of 22 per 1,000 (95% CI 6–76; I^2^ = 92%), Eastern Mediterranean of 15 per 1,000 (95% CI 10–22; I^2^ = 95%), and the South East Asian of 21 per 1,000 (95% CI 13–32; I^2^ = 98%). The European region, represented only by the study of Atalay et al. (30) performed in Turkey, had a prevalence of 23 per 1,000 individuals (95% CI 18–30).

### Sensitivity analyses

Meta-regression analyses explored potential sources of heterogeneity and effect-modifications influencing the prevalence of RHD, covering both the overall prevalence and prevalence specific to continents and countries (Supplementary Table S5). The initial and final screening years did not yield statistically significant variations in prevalence. In contrast, age and GNI per capita exhibited a positive association with latent RHD prevalence. The supplement presents results for both the leave-one-out sensitivity analysis, which evaluates the influence of individual studies on the pooled estimated prevalence, and the funnel plots, which indicated asymmetry, possibly due to high study heterogeneity (Supplementary Figure S8, S9). The leave-one-out sensitivity analysis confirms the consistency of our results.

### Quality assessment

Out of the 58 studies reviewed, two were found to exhibit a moderate risk of bias, while the remaining 56 studies were deemed to have a low risk of bias. All 58 studies collected data directly from the participants, and the instruments employed to assess the variables of interest were considered suitable. Given that most of the screenings took place in school settings, non-response bias was minimal. Among the 58 studies, only 14 incorporated a form of randomization in sample selection, by randomizing the schools where the screening occurred. For more detailed information on the ten risks of bias criteria across all studies, refer to the supplement (Supplementary Table S4).

## Discussion

We conducted a comprehensive systematic review and meta-analysis to determine the prevalence of RHD, analyzing data from 58 studies encompassing a total of 215,552 children and adolescents. The overarching prevalence of RHD was found to be 24 per 1,000 individuals, and we identified a consistent prevalence in all endemic areas. This meta-analysis differs from prior work by focusing exclusively on echocardiographic screening studies, examining RHD severity and morphological features, and addressing sex disparities in latent RHD. By directly comparing prevalence across subgroups, we confirmed previous knowledge and highlighted vulnerable populations—(i) females, (ii) individuals in rural areas, (iii) students in public schools, and (iv) low-income households. Additionally, latent RHD prevalence exceeded twice in patients aged 10 years or older compared to younger individuals.

Chronic RHD exhibits a higher prevalence among females, with at least a 2:1 ratio (5, 9, 10). Previous studies including patients with rheumatic mitral stenosis, a late stage of RHD, reveal an even higher female predominance, exceeding 80% (10, 79). However, ARF, the primary precursor to RHD, has not been reported as more prevalent in female children and adolescents; most studies suggest a 1:1 ratio (4). Our study extends the findings of a prior meta-analysis, which, via univariate meta-regression, revealed an association between female sex and latent RHD diagnosis (80). In our investigation, this finding was confirmed through a first direct comparison of latent RHD prevalence across sexes, with a ratio female/male of 1·4:1. In summary, current literature supports that (i) ARF has approximately a 1:1 female/male ratio, our meta-analysis showed that it is (ii) 1·4:1 in latent RHD and is well-known that (iii) chronic it is at least 2:1. These findings suggest a tendency towards a higher prevalence of RHD progression in females.

Environmental factors may contribute to the increased risk in females, given the more frequent role of women in caring for children and younger siblings outside their household, which may expose them to a greater risk of group A streptococcus infections (4, 81). In addition, females typically are also more susceptible to autoimmune conditions, which may contribute to these outcomes (82). A recent study conducted proteomic analysis on 30 cardiac valves from patients without RHD and compared them to valves affected by RHD (5). This investigation revealed a higher presence of prothymosin-alpha in RHD-related valve pathologies (5). Notably, this protein, which plays an important pathogenetic role in streptococcal antigen presentation by HLA molecules and CD8 lymphocyte activation, is linked to estrogen receptor alpha activity, suggesting its potential role as a regulator of the female predisposition to developing RHD (5).

RHD exerts a greater burden on low-to-middle-income nations (14). This may be secondary to superior healthcare in middle-to-high-income nations, including prompt treatment for streptococcal sore throats more widespread implementation of secondary prophylaxis with penicillin, and better living conditions that avoid overcrowding (81). Disparities in healthcare support can also be observed within regions of the same country. Our study revealed, by direct comparison, a heightened prevalence of latent RHD among children from rural areas, those attending public schools, and those from low-income families, all of whom exhibit increased health vulnerability. The study with the highest latent RHD prevalence included African refugees residing in Italy and unveiled a prevalence of 21%, which is tenfold greater than the overall prevalence in our analysis (69). This can indicate the environmental vulnerability associated with the disease and immigrant population.

The WHO first proposed echocardiographic screening criteria for RHD in 2005, introducing the concepts of definite, probable, and possible disease based on echocardiographic findings and epidemiological factors such as residence in endemic areas or a history of ARF. In 2012, the WHF released updated criteria (19), defining only borderline and definite RHD, based solely on echocardiographic parameters (see Supplementary Appendix). In our analysis, the prevalence by WHO criteria (eight studies) was 24 per 1,000 (95% CI 13–40; I^2^ = 97%), and by WHF criteria (46 studies) 25 per 1,000 (95% CI 19–32; I^2^ = 98%), with no significant difference. However, rates varied among studies, and Spitzer et al. (66) did a direct comparison in a Peruvian cohort and found prevalence rates of 19.7/1,000 (WHO) vs. 3.9/1,000 (2012 WHF). Most recently, the 2023 WHF classification introduced four stages (A–D) reflecting valve morphology and regurgitation severity (15). This staging recognizes RHD as a spectrum, enhancing the understanding of disease progression. Nevertheless, none of the included studies were performed after the adoption of the new WHF criteria.

Latent RHD was categorized into two groups according to 2012 WHF criteria: definite and borderline (19). Within the definite category, there are further subdivisions, namely mild, moderate, and severe, each associated with distinct disease progression patterns and outcomes. Moderate and severe RHD exhibit a higher propensity for disease progression and are linked to increased mortality (83). One study followed latent RHD patients for over one year, revealing that, during the follow-up, 40% of those with moderate to severe definite RHD experienced disease progression, and 10% succumbed to the condition (83). A meta-analysis on disease progression reported a 7.5% progression rate in definite RHD, with 60% remaining stable; however, one limitation was the variability in follow-up durations across studies (80). Therefore, a large amount of latent RHD patients improve without any treatment. In our pooled analysis, 41% of individuals with definite RHD had moderate-severe disease. The overall prevalence of definite RHD by WHF among screened children is 9 cases per 1,000, and with moderate-severe definite RHD at 41%, it is plausible that more than 3 children out of every 1,000 in endemic regions may have a more severe form of the disease.

Definite RHD could benefit from a screening program: (i) it typically manifests later in life, stemming mainly from childhood ARF (4); (ii) there is a disease progression, advancing from borderline latent RHD to definite latent RHD and ultimately clinical RHD (80); (iii) Current evidence supports that secondary prophylaxis can prevent disease progression (11), and (iv) sensitive diagnostic examinations as echocardiogram are available for detection (12). Efforts to combat RHD could greatly benefit from the identification of a biomarker. The Leducq Foundation is currently funding multi-center research groups aimed at discovering a biomarker for ARF that could be utilized for effective screening. Nonetheless, some challenges persist, such as the need for specialized echocardiogram interpretation, more studies on secondary prophylaxis, and the necessity for further data to determine the optimal age for screening initiation, as the timing of maximum treatment effectiveness remains uncertain. Our meta-analysis unveiled a higher prevalence of latent RHD in children aged 10 years and older when compared with those younger than 10 years (Supplementary Figure S6). This outcome aligns with expectations, given that the disease exhibits progressive development over time, with a significant proportion of ARF cases occurring between the ages of 5 and 15 years.

Our study contributes to the discussion on public health strategies for RHD prevention, such as echocardiographic screening and subsequent penicillin prophylaxis. Before these approaches can be implemented as public policy, further studies are needed to assess cost-effectiveness and to compare screened and treated groups with unscreened populations. Based on our findings, initial screening efforts could focus on high-risk settings, such as rural areas, low-income families, and children attending public schools, who are likely to benefit the most. Additionally, a promising strategy to provide region specific data and further understand disease burden is the development of high-quality RHD databases, such as the ARGI from Egypt (84).

Two previous meta-analyses (2014 and 2019) examined the global prevalence of latent RHD (80, 85). However, the current meta-analysis provides a more focused and comprehensive approach by narrowing the scope to echocardiogram-based screening studies in endemic regions. This strategy allowed us to delve deeper into at-risk groups, identifying higher susceptibility among females, public school children, rural residents, and individuals from low-income families. Additionally, we assessed prevalence patterns based on echocardiogram criteria, mitral lesion types, and the prevalence of moderate and severe cases. Importantly, our systematic search incorporated 24 new studies, screening over 85,000 individuals, significantly expanding the evidence base since the last global meta-analysis. This updated and detailed perspective highlights emerging trends and provides critical insights to guide targeted interventions and policy development in high-burden regions.

Our study has limitations. As a worldwide systematic review, we utilized data with varying inclusion criteria, ethnicities, baseline characteristics, and risk factors. While these factors contributed to a broader result, they also increased heterogeneity. To mitigate this, we only included studies that used echocardiogram as a screening tool due to its high sensitivity compared to other methods. Nevertheless, the consistency of overall prevalence across the endemic areas despite different clinical and geographic settings suggests that the current results are reproducible. Furthermore, we scrutinized the findings by constructing a meta-regression with several potential confounding or modifier variables. Ultimately, the heterogeneity may simply be secondary to different regional prevalences between studies.

## Conclusion

The present study reveals a higher prevalence of RHD in females, consistent with patterns observed in chronic RHD. By highlighting the early-stage female predominance in latent RHD, our study provides support for the concept that gender disparity in RHD emerges at an early stage, preceding the onset of heart valve damage. Moreover, there is a higher prevalence in children from rural areas, public schools, and low-income families, emphasizing the need for targeted interventions in these vulnerable populations.

## Data Availability

The original contributions presented in the study are included in the article/Supplementary Material, further inquiries can be directed to the corresponding author.
