# Supplementary material for: Global prevalence and sex differences in rheumatic heart disease: a systematic review and updated meta-analysis
Source: Front Cardiovasc Med. 2025 Nov 6;12:1615158. doi: 10.3389/fcvm.2025.1615158 (PMC12631635; doi:10.3389/fcvm.2025.1615158)
Supplement: Supplementary file 1 [file Datasheet1.pdf]

Supplementary Material

TABLE OF CONTENTS:

S. Table 1: Search strategies

S. Table 2: Excluded studies

S. Table 3: Characteristics of the included studies

S. Table 4: Risk of Bias of included studies

S. Table 5: Meta regression

S. Figure 1: Prevalence of definite RHD

S. Figure 2: Prevalence of borderline RHD

S. Figure 3: Prevalence of Latent RHD by WHO regions

S. Figure 4: Prevalence of Latent RHD by continent

S. Figure 5: Comparison between boys and girls by definite RHD

S. Figure 6: Prevalence of latent RHD by age

S. Figure 7: Table and figures – prevalence of WHF diagnostic criteria

S. Figure 8: Leave-one-out analysis

S. Figure 9: Funnel plot of the primary outcome

REFERENCES

S. Table 1: Search strategies

Data base	Search strategy
**EMBASE**	Rheumatic AND (Heart OR Valvular OR Cardiopathy OR Cardiomyopathy) AND (Prevalence OR Screening OR Surveillance OR Epidemiology) AND (Child OR Adolescent OR School OR Children OR teenager)
**PubMed**	Rheumatic AND (Heart OR Valvular OR Cardiopathy OR Cardiomyopathy) AND (Prevalence OR Screening OR Surveillance OR Epidemiology) AND (Child OR Adolescent OR School OR Children OR teenager)
**Cochrane**	Rheumatic AND (Heart OR Valvular OR Cardiopathy OR Cardiomyopathy) AND (Prevalence OR Screening OR Surveillance OR Epidemiology) AND (Child OR Adolescent OR School OR Children OR teenager)
**Lilacs**	Rheumatic AND (Heart OR Valvular OR Cardiopathy OR Cardiomyopathy) AND (Prevalence OR Screening OR Surveillance OR Epidemiology) AND (Child OR Adolescent OR School OR Children OR teenager)

S. Table 2: Excluded studies

Study	Exclusion criteria
Ahmed, 2005[1]<br>Anabwani, 1996[2]<br>Fakakonikaetau, 2016[3]<br>Farrag, 2014[4]<br>Kwok, 2014[5]<br>Rossi, 2014[6]<br>Viali, 2016[7]<br>Yildz, 2016[8]<br>Min, 2023[9]	Conference abstract only
Sriharibabu, 2014[10]<br>Zhimin, 2006[11]	Reported RHD in adults
Longo-Mbenza,1998[12]<br>Zaman, 2015[13]<br>Amade, 2023[14]	Echocardiogram only in suspected cases
Schaffer, 2003[15]	Not endemic country
Zuhlke, 2016[16]	Patients with prior RHD diagnosis
Sanyahumbi, 2019[17]<br>Hempenstall, 2021[18]<br>Laudari, 2019[19]<br>Mirabel, 2015[20]<br>Shrestha, 2021[21]<br>Zuhlke, 2016[22]<br>Remenyi, 2020[23]<br>Roberts, 2014[24]	Overlapping population with included studies
Miranda, 2014[25]<br>Reeves, 2011[26]	Small sample size

S. Table 3: Characteristics of included studies

Study	Risk of Bias	Country	Continent	Population	Age range	Sample size	First screening year	Last screening year	Diagnostic criteria
Ali, 2018	Low	Sudan	Africa	School children	10-15	4498	2015	2016	WHF
Ali, 2018	Low	Sudan	Africa	School children	11-15	2129	2016	2018	WHF
Ali, 2022	Low	Sudan	Africa	School children	10-18	4572	2020	2021	WHF
Allen, 2017	Low	Samoa	Oceania	School children	5-17	11434	2013	2015	WHF
Atalay, 2019	Low	Turkey	Asia	School children	5-18	2550	2017	2018	WHF
Baroux, 2013	Low	New Caledonia	Oceania	School children	7-14	12803	2008	2010	Other
Beaton, 2012	Low	Uganda	Africa	School children	5-16	4869	2010	2010	WHO
Beaton, 2015	Low	Uganda	Africa	School children		4773			WHF
Bertaina, 2017	Low	New Caledonia	Oceania	School children	9-10	8684	2012	2014	WHF
Bhaya, 2010	Low	India	Asia	School children	6-15	1059	2007	2008	WHO
Bhaya, 2019	Low	India	Asia	School children	11-15	3000			WHF
Campanale, 2017	Low	Madagascar	Africa	School children	5-19	522	2013	2015	WHF
Chatard, 2020	Low	New Caledonia	Oceania	School children	16	1530	2017	2018	WHF
Chillo, 2023	Low	Tanzania	Africa	School children	5-16	4436	2018	2019	WHF
Choudhary, 2021	Low	India	Asia	School children	10-15	3000	2015	2018	WHF
Colquhoun, 2014	Low	Fiji	Oceania	School children	5-14	1666	2009	2010	WHO
Condemi, 2019	Moderate	Imigrants*	Africa	Community	12-18	639	2016	2018	WHF
Corsenac, 2016	Low	New Caledonia	Oceania	School children	9-10	7002	2012	2013	WHF
Cramp, 2012	Low	New Zeland	Oceania	School children	5-18	685	2009	2009	WHO
Davis, 2018	Low	timor leste	Asia	School children	5-20	1365			WHF
Ekure, 2019	Low	Nigeria	Africa	School children	5-16	4107	2016	2017	WHF

Engel, 2015	Low	Ethiopia	Africa	School Children	5-20	2000	2010	2013	WHF
Engelman, 2016	Low	Fiji	Oceania	School children	5-15	2004	2012	2013	WHF
Francis, 2020	Low	Australia	Oceania	School children	5-20	613	2018	2018	WHF
Francis, 2021	Low	Timor leste AND Australia	Asia AND Oceania	Community	5-20	2573	2018	2018	WHF
Francis, 2023	Low	Timor leste AND Australia	Asia AND Oceania	School Children and Community	5-20	3329	2019	2019	WHF
Godown, 2015	Low	Uganda	Africa	School children	5-17	4773			WHF
Hosseini, 2022	Low	Iran	Asia	School children	6-18	15130	2017	2018	WHF
Huang, 2017	Low	American Samoa	Oceania	School children	5-18	1058			WHF
Hunter, 2021	Low	South Africa	Africa	School children	13-19	5255	2014	2015	WHF
Johannsen, 2021	Moderate	Kenya and Cameroon	Africa	School children	8-18	907			WHF
Kaltenborn, 2023	low	Ethiopia	Africa	School children	6-18	6631	2019	2022	Other
Kane, 2012	Low	Senegal	Africa	School children	5-18	2004	2010	2010	Other
Karki, 2021	Low	Nepal	Asia	School children	5-12	3973	2012	2014	WHF
Kazahura, 2021	Low	Tanzania	Africa	School children	7-18	949	2019	2019	WHF
Kotit, 2017	Low	Egypt	Africa	School children	5-15	3062	2009	2014	WHF
Kumari, 2013	Low	India	Asia	School children	5-16	4213	2011	2011	WHO
Lu, 2015	Low	Uganda	Africa	School children	5-17	1439			WHF
Marijon, 2007	Low	Cambodia	Asia	School children	6-16	3677	2001	2002	Other
Marijon, 2009	Low	Mozambique	Africa	School children	6-17	2170	2005	2005	WHO
Mirabel, 2015	Low	New Caledonia	Oceania	School children		1217	2013	2013	WHF
Mucumbitsi, 2017	Low	Rwanda	Africa	School children	6-16	2501			WHF
Musuku, 2018	Low	Zambia	Africa	School children	10-18	1037	2014	2015	WHF
Nascimento, 2018	Low	Brazil	Latin America	School children	10-18	12048	2014	2017	WHF
Ngaïdé, 2015	Low	Senegal	Africa	School children	5-18	2019	2011	2011	WHF

Paar, 2010	Low	Nicaragua	Latin Amercia	Community	5-15	3150	2006	2009	WHO
Ploutz, 2015	Low	Uganda	Africa	School children	5-17	956	2014	2014	WHF
Remenyi, 2019	Low	Timor-Leste	Asia	School children	5-20	1365	2016	2016	WHF
Roberts, 2015	Low	Australia	Oceania	Community	5-15	3946	2008	2010	WHF
Sanyahumbi, 2016	Low	Malawi	Africa	School Children and Community	5-16	1450	2014	2014	WHF
Saxena, 2011	Low	India	Asia	School children	5-15	6270	2008	2010	WHO
Saxena, 2017	Low	India	Asia	School children	5-15	16294	2010	2013	WHF
Scheel, 2018	Low	Uganda	Africa	Community	5-20	1470			WHF
Shrestha, 2016	Low	Nepal	Asia	School children	5-15	5178	2012	2014	WHF
Splitzer, 2015	Low	Peru	Latin America	School children	5-16	1023	2014	2014	WHO
Voleti, 2021	Low	Palau	Oceania	School children	6-15	632			WHF
Webb, 2011	Low	New Zeland	Oceania	Community	10-13	1142			WHO
Yadeta, 2016	Low	Ethiopia	Africa	School children	6-18	3238	2013	2014	WHF

S. Table 4: Risk of Bias by Hoy

study	Criteria 1	Criteria 2	Criteria 3	Criteria 4	Criteria 5	Criteria 6	Criteria 7	Criteria 8	Criteria 9	Criteria 10	overall
Ali, 2018	1	1	1	1	1	1	1	1	1	1	10
Chillo, 2023	1	1	1	1	1	1	1	1	1	1	10
Francis, 2020	1	1	1	1	1	1	1	1	1	1	10
Hosseini, 2022	1	1	1	1	1	1	1	1	1	1	10
Karki, 2021	1	1	1	1	1	1	1	1	1	1	10
Lu, 2015	1	1	1	1	1	1	1	1	1	1	10
Marijon, 2007	1	1	1	1	1	1	1	1	1	1	10
Marijon, 2009	1	1	1	1	1	1	1	1	1	1	10
Musuku, 2018	1	1	1	1	1	1	1	1	1	1	10
Paar, 2010	1	1	1	1	1	1	1	1	1	1	10
Saxena, 2011	1	1	1	1	1	1	1	1	1	1	10
Shrestha, 2016	1	1	1	1	1	1	1	1	1	1	10
Splitzer, 2015	1	1	1	1	1	1	1	1	1	1	10
Ali, 2022	1	1	1	0	1	1	1	1	1	1	9
Allen, 2017	1	1	0	1	1	1	1	1	1	1	9
Baroux, 2013	1	1	0	1	1	1	1	1	1	1	9
Beaton, 2012	1	1	0	1	1	1	1	1	1	1	9
Bhaya, 2010	1	1	0	1	1	1	1	1	1	1	9
Bhaya, 2019	1	1	0	1	1	1	1	1	1	1	9
Chatard, 2020	1	1	0	1	1	1	1	1	1	1	9
Colquhoun, 2014	1	1	0	1	1	1	1	1	1	1	9
Davis, 2018	1	1	0	1	1	1	1	1	1	1	9

Ekure, 2019	1	1	0	1	1	1	1	1	1	1	9
Engel, 2015	0	1	1	1	1	1	1	1	1	1	9
Francis, 2021	1	1	0	1	1	1	1	1	1	1	9
Gemechu, 2017	1	1	0	1	1	1	1	1	1	1	9
Huang, 2017	1	1	0	1	1	1	1	1	1	1	9
Kane, 2012	1	1	0	1	1	1	1	1	1	1	9
Kazahura, 2021	1	1	0	1	1	1	1	1	1	1	9
Mucumbitsi, 2017	1	1	0	1	1	1	1	1	1	1	9
Ngaïdé, 2015	1	1	0	1	1	1	1	1	1	1	9
Remenyi, 2019	1	1	0	1	1	1	1	1	1	1	9
Roberts, 2015	1	1	0	1	1	1	1	1	1	1	9
Sanyahumbi, 2016	1	1	0	1	1	1	1	1	1	1	9
Saxena, 2017	1	1	0	1	1	1	1	1	1	1	9
Webb, 2011	1	1	0	1	1	1	1	1	1	1	9
Ali, 2018	1	1	0	0	1	1	1	1	1	1	8
Atalay, 2019	1	1	0	1	1	1	1	1	1	0	8
Beaton, 2015	1	1	0	1	1	1	1	0	1	1	8
Bertaina, 2017	1	1	0	1	1	1	1	1	1	0	8
Campanale, 2017	1	1	0	1	1	1	1	0	1	1	8
Choudhary, 2021	1	1	0	1	1	1	1	1	1	0	8
Corsenac, 2016	1	1	0	0	1	1	1	1	1	1	8
Engelman, 2016	1	0	0	1	1	1	1	1	1	1	8
Francis, 2023	1	0	0	1	1	1	1	1	1	1	8
Godown, 2015	1	0	0	1	1	1	1	1	1	1	8
Hunter, 2021	1	1	0	1	1	1	1	1	1	0	8
Kaltenborn, 2023	1	1	0	1	1	1	1	0	1	1	8

Kumari, 2013	0	1	0	1	1	1	1	1	1	1	8
Nascimento, 2018	1	1	0	1	1	1	1	0	1	1	8
Scheel, 2018	0	1	0	1	1	1	1	1	1	1	8
Voleti, 2021	1	1	0	1	1	1	1	0	1	1	8
Yadeta, 2016	1	1	0	1	1	1	1	1	1	0	8
Kotit, 2017	0	0	0	1	1	1	1	1	1	1	7
Mirabel, 2015	1	0	0	1	1	1	1	1	1	0	7
Ploutz, 2015	1	1	0	1	1	1	0	0	1	1	7
Johannsen, 2021	1	0	0	1	1	1	1	0	1	0	6
Condemi, 2019	0	0	0	0	1	1	1	1	1	0	5

S. Table 5: Meta-Regression

Outcome	Predictor		Beta (SE)	Intercept (SE)	p-value
Age	10 years old or younger		-0·821 (0·27)	-0·657 (0·17)	**0·0027**
Continent	WHO region			-3·711 (0·18)	
		Eastern Mediterranean	-0·483 (0.41)		0·29
		European	0·040 (0·84)		0·99
		East Asian	-0·151 (0·30)		0·74
		The Americas	0·035 (0·52)		0·97
		Western Pacific	0·300 (0·27)		0·19
Continent	Mean years of schooling		0·005 (0·00)	-4·040 (0·23)	0·10
Continent	GNI per capita		0·000 (0·00)	-3·894 (0·13)	**0·0084**
Continent	Expected years of schooling		0·000 (0·00)	-3·727 (0·31)	0·90
Latent RHD	Year of publication		0·026 (0·03)	-56·10 (65·6)	0·40
	Last year screening		0·040 (0·03)	-85·26 (59·7)	0·17
	First year screening		0·039 (0·03)	-81·86 (59·8)	0·19

S. Figure 1: Prevalence of definite RHD

Study	Events	Total	Proportion [95% CI]
Bertaina, 2017	2	8684	0.0002 [0.0000; 0.0008]
Ekure, 2019	2	4107	0.0005 [0.0001; 0.0018]
Splitzer, 2015	1	1023	0.0010 [0.0000; 0.0054]
Karki, 2021	4	3973	0.0010 [0.0003; 0.0026]
Paar, 2010	5	3150	0.0016 [0.0005; 0.0037]
Mucumbitsi, 2017	4	2501	0.0016 [0.0004; 0.0041]
Beaton, 2012	8	4869	0.0016 [0.0007; 0.0032]
Saxena, 2017	32	16294	0.0020 [0.0013; 0.0028]
Hosseini, 2022	30	15130	0.0020 [0.0013; 0.0028]
Musuku, 2018	3	1037	0.0029 [0.0006; 0.0084]
Roberts, 2015	18	3946	0.0046 [0.0027; 0.0072]
Ngaïdé, 2015	10	2019	0.0050 [0.0024; 0.0091]
Nascimento, 2018	63	12048	0.0052 [0.0040; 0.0067]
Scheel, 2018	8	1470	0.0054 [0.0024; 0.0107]
Cramp, 2012	4	685	0.0058 [0.0016; 0.0149]
Sanyahumbi, 2016	10	1450	0.0069 [0.0033; 0.0126]
Shrestha, 2016	36	5178	0.0070 [0.0049; 0.0096]
Engelman, 2016	14	2004	0.0070 [0.0038; 0.0117]
Webb, 2011	8	1142	0.0070 [0.0030; 0.0138]
Corsenac, 2016	53	7002	0.0076 [0.0057; 0.0099]
Campanale, 2017	4	522	0.0077 [0.0021; 0.0195]
Marijon, 2009	17	2170	0.0078 [0.0046; 0.0125]
Colquhoun, 2014	14	1666	0.0084 [0.0046; 0.0141]
Baroux, 2013	114	12803	0.0089 [0.0074; 0.0107]
Hunter, 2021	49	5255	0.0093 [0.0069; 0.0123]
Ali, 2018	20	2129	0.0094 [0.0057; 0.0145]
Beaton, 2015	47	4773	0.0098 [0.0072; 0.0131]
Ali, 2018	45	4498	0.0100 [0.0073; 0.0134]
Allen, 2017	115	11434	0.0101 [0.0083; 0.0121]
Godown, 2015	52	4773	0.0109 [0.0081; 0.0143]
Kane, 2012	22	2004	0.0110 [0.0069; 0.0166]
Ploutz, 2015	11	956	0.0115 [0.0058; 0.0205]
Mirabel, 2015	15	1217	0.0123 [0.0069; 0.0202]
Chillo, 2023	59	4436	0.0133 [0.0101; 0.0171]
Yadeta, 2016	44	3238	0.0136 [0.0099; 0.0182]
Francis, 2023	47	3329	0.0141 [0.0104; 0.0187]
Voleti, 2021	9	632	0.0142 [0.0065; 0.0269]
Atalay, 2019	39	2550	0.0153 [0.0109; 0.0208]
Engel, 2015	33	2000	0.0165 [0.0114; 0.0231]
Kazahura, 2021	17	949	0.0179 [0.0105; 0.0285]
Davis, 2018	25	1365	0.0183 [0.0119; 0.0269]
Kotit, 2017	60	3062	0.0196 [0.0150; 0.0252]
Marijon, 2007	79	3677	0.0215 [0.0170; 0.0267]
Chatard, 2020	37	1530	0.0242 [0.0171; 0.0332]
Condemi, 2019	17	639	0.0266 [0.0156; 0.0423]
Choudhary, 2021	88	3000	0.0293 [0.0236; 0.0360]
Bhaya, 2019	90	3000	0.0300 [0.0242; 0.0367]
Francis, 2021	82	2573	0.0319 [0.0254; 0.0394]
Lu, 2015	52	1439	0.0361 [0.0271; 0.0471]
Huang, 2017	41	1058	0.0388 [0.0280; 0.0522]
Gemechu, 2017	36	898	0.0401 [0.0282; 0.0551]
Francis, 2020	32	613	0.0522 [0.0360; 0.0729]
Kaltenborn, 2023	425	6631	0.0641 [0.0583; 0.0703]
**Total (95% CI)**		**198531**	**0.0087 [0.0065; 0.0118]**

Heterogeneity: $Tau^2 = 1.1776$; $Chi^2 = 1895.80$, df = 52 (P = 0); $I^2 = 97\%$

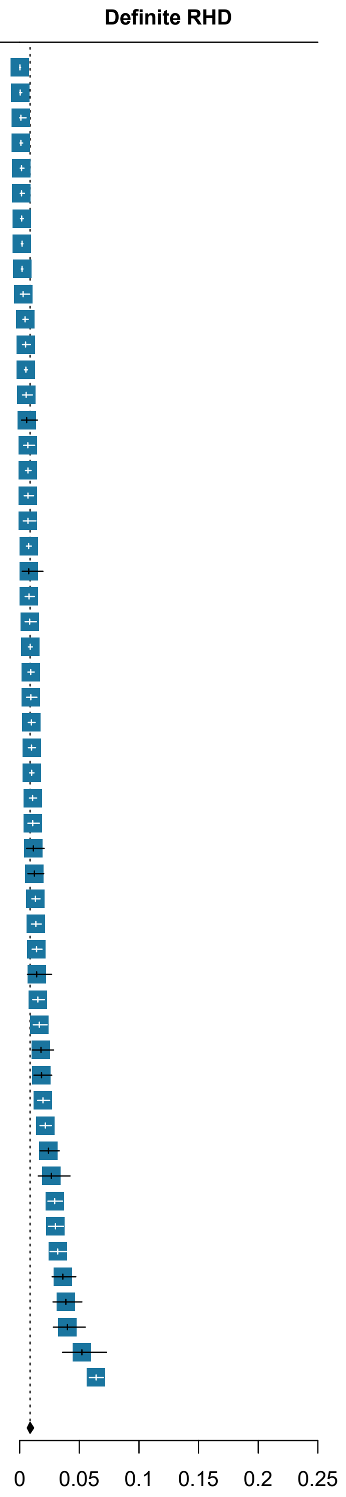

S. Figure 2: Prevalence of Borderline RHD

Study	Events	Total	Proportion [95% CI]
Bertaina, 2017	15	8684	0.002 [0.001; 0.003]
Ekure, 2019	9	4107	0.002 [0.001; 0.004]
Splitzer, 2015	3	1023	0.003 [0.001; 0.009]
Shrestha, 2016	17	5178	0.003 [0.002; 0.005]
Yadeta, 2016	15	3238	0.005 [0.003; 0.008]
Ali, 2018	21	4498	0.005 [0.003; 0.007]
Mucumbitsi, 2017	13	2501	0.005 [0.003; 0.009]
Saxena, 2017	93	16294	0.006 [0.005; 0.007]
Campanale, 2017	3	522	0.006 [0.001; 0.017]
Corsenac, 2016	44	7002	0.006 [0.005; 0.008]
Karki, 2021	26	3973	0.007 [0.004; 0.010]
Hosseini, 2022	113	15130	0.007 [0.006; 0.009]
Ali, 2018	16	2129	0.008 [0.004; 0.012]
Bhaya, 2019	23	3000	0.008 [0.005; 0.011]
Atalay, 2019	20	2550	0.008 [0.005; 0.012]
Allen, 2017	92	11434	0.008 [0.006; 0.010]
Chillo, 2023	36	4436	0.008 [0.006; 0.011]
Choudhary, 2021	27	3000	0.009 [0.006; 0.013]
Musuku, 2018	10	1037	0.010 [0.005; 0.018]
Colquhoun, 2014	18	1666	0.011 [0.006; 0.017]
Ngaïdé, 2015	23	2019	0.011 [0.007; 0.017]
Kotit, 2017	35	3062	0.011 [0.008; 0.016]
Roberts, 2015	62	3946	0.016 [0.012; 0.020]
Kazahura, 2021	15	949	0.016 [0.009; 0.026]
Davis, 2018	23	1365	0.017 [0.011; 0.025]
Remenyi, 2019	23	1365	0.017 [0.011; 0.025]
Chatard, 2020	26	1530	0.017 [0.011; 0.025]
Scheel, 2018	29	1470	0.020 [0.013; 0.028]
Hunter, 2021	104	5255	0.020 [0.016; 0.024]
Gemechu, 2017	19	898	0.021 [0.013; 0.033]
Engelman, 2016	43	2004	0.021 [0.016; 0.029]
Francis, 2021	60	2573	0.023 [0.018; 0.030]
Francis, 2023	86	3329	0.026 [0.021; 0.032]
Sanyahumbi, 2016	39	1450	0.027 [0.019; 0.037]
Voleti, 2021	17	632	0.027 [0.016; 0.043]
Francis, 2020	17	613	0.028 [0.016; 0.044]
Beaton, 2015	133	4773	0.028 [0.023; 0.033]
Mirabel, 2015	34	1217	0.028 [0.019; 0.039]
Godown, 2015	140	4773	0.029 [0.025; 0.035]
Ploutz, 2015	32	956	0.033 [0.023; 0.047]
Nascimento, 2018	478	12048	0.040 [0.036; 0.043]
Huang, 2017	96	1058	0.091 [0.074; 0.110]
Lu, 2015	140	1439	0.097 [0.082; 0.114]
Condemi, 2019	120	639	0.188 [0.158; 0.220]
**Total (95% CI)**		**160765**	**0.013 [0.010; 0.018]**

Heterogeneity: $Tau^2 = 0.9558$; $Chi^2 = 2267.74$, df = 43 (P = 0); $I^2 = 98\%$

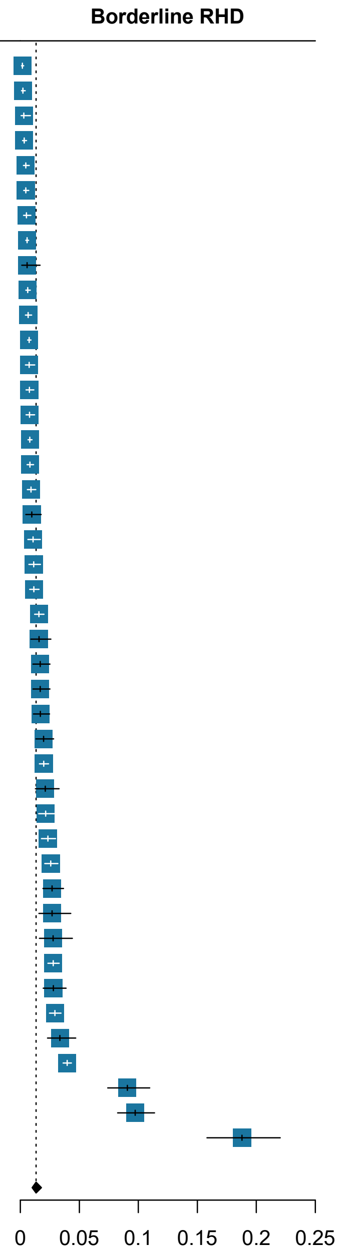

S. Figure 3: Prevalence by WHO regions

Study or Subgroup	Events	Total	Proportion [95% CI]
**Western Pacific**			
Bertaina, 2017	49	8684	0.0056 [0.0042; 0.0075]
Baroux, 2013	114	12803	0.0089 [0.0074; 0.0107]
Corsenac, 2016	97	7002	0.0139 [0.0112; 0.0169]
Allen, 2017	207	11434	0.0181 [0.0157; 0.0207]
Colquhoun, 2014	32	1666	0.0192 [0.0132; 0.0270]
Roberts, 2015	80	3946	0.0203 [0.0161; 0.0252]
Marijon, 2007	79	3677	0.0215 [0.0170; 0.0267]
Engelman, 2016	57	2004	0.0284 [0.0216; 0.0367]
Mirabel, 2015	49	1217	0.0403 [0.0299; 0.0529]
Voleti, 2021	26	632	0.0411 [0.0270; 0.0597]
Chatard, 2020	63	1530	0.0412 [0.0318; 0.0524]
Cramp, 2012	30	685	0.0438 [0.0297; 0.0619]
Webb, 2011	59	1142	0.0517 [0.0396; 0.0661]
Francis, 2020	49	613	0.0799 [0.0597; 0.1043]
Francis, 2021	45	553	0.0814 [0.0600; 0.1074]
Francis, 2023	71	762	0.0932 [0.0735; 0.1161]
Huang, 2017	137	1058	0.1295 [0.1098; 0.1512]
**Total (95% CI)**		**59408**	**0.0319 [0.0214; 0.0474]**
Heterogeneity: $Tau^2 = 0.7255$; $Chi^2 = 970.71$, df = 16 ($P < 0.01$); $I^2 = 98\%$			
**The Americas**			
Splitzer, 2015	4	1023	0.0039 [0.0011; 0.0100]
Nascimento, 2018	541	12048	0.0449 [0.0413; 0.0488]
Paar, 2010	150	3150	0.0476 [0.0404; 0.0556]
**Total (95% CI)**		**16221**	**0.0218 [0.0060; 0.0763]**
Heterogeneity: $Tau^2 = 1.2627$; $Chi^2 = 25.1$, df = 2 ($P < 0.01$); $I^2 = 92\%$			
**Eastern Mediterranean**			
Hosseini, 2022	143	15130	0.0095 [0.0080; 0.0111]
Ali, 2022	47	4572	0.0103 [0.0076; 0.0136]
Ali, 2018	66	4498	0.0147 [0.0114; 0.0186]
Ali, 2018	36	2129	0.0169 [0.0119; 0.0233]
Kotit, 2017	95	3062	0.0310 [0.0252; 0.0378]
**Total (95% CI)**		**29391**	**0.0149 [0.0101; 0.0218]**
Heterogeneity: $Tau^2 = 0.1792$; $Chi^2 = 88.74$, df = 4 ($P < 0.01$); $I^2 = 95\%$			
**African**			
Ekure, 2019	11	4107	0.0027 [0.0013; 0.0048]
Mucumbitsi, 2017	17	2501	0.0068 [0.0040; 0.0109]
Marijon, 2009	17	2170	0.0078 [0.0046; 0.0125]
Kane, 2012	22	2004	0.0110 [0.0069; 0.0166]
Musuku, 2018	13	1037	0.0125 [0.0067; 0.0213]
Campanale, 2017	7	522	0.0134 [0.0054; 0.0274]
Beaton, 2012	72	4869	0.0148 [0.0116; 0.0186]
Ngaïdé, 2015	33	2019	0.0163 [0.0113; 0.0229]
Yadeta, 2016	59	3238	0.0182 [0.0139; 0.0234]
Chillo, 2023	95	4436	0.0214 [0.0174; 0.0261]
Scheel, 2018	37	1470	0.0252 [0.0178; 0.0345]
Hunter, 2021	153	5255	0.0291 [0.0247; 0.0340]
Engel, 2015	61	2000	0.0305 [0.0234; 0.0390]
Kazahura, 2021	32	949	0.0337 [0.0232; 0.0473]
Sanyahumbi, 2016	49	1450	0.0338 [0.0251; 0.0444]
Beaton, 2015	180	4773	0.0377 [0.0325; 0.0435]
Godown, 2015	192	4773	0.0402 [0.0348; 0.0462]
Ploutz, 2015	43	956	0.0450 [0.0327; 0.0601]
Johannsen, 2021	52	907	0.0573 [0.0431; 0.0745]
Gemechu, 2017	55	898	0.0612 [0.0465; 0.0790]
Kaltenborn, 2023	425	6631	0.0641 [0.0583; 0.0703]
Lu, 2015	192	1439	0.1334 [0.1163; 0.1521]
**Total (95% CI)**		**58404**	**0.0238 [0.0166; 0.0340]**
Heterogeneity: $Tau^2 = 0.7428$; $Chi^2 = 859.43$, df = 21 ($P < 0.01$); $I^2 = 98\%$			
**South East Asian**			
Karki, 2021	30	3973	0.0076 [0.0051; 0.0108]
Kumari, 2013	32	4213	0.0076 [0.0052; 0.0107]
Saxena, 2017	125	16294	0.0077 [0.0064; 0.0091]
Shrestha, 2016	53	5178	0.0102 [0.0077; 0.0134]
Saxena, 2011	128	6270	0.0204 [0.0171; 0.0242]
Francis, 2023	62	2567	0.0242 [0.0186; 0.0309]
Davis, 2018	48	1365	0.0352 [0.0260; 0.0464]
Bhaya, 2019	113	3000	0.0377 [0.0311; 0.0451]
Choudhary, 2021	115	3000	0.0383 [0.0317; 0.0458]
Francis, 2021	97	2020	0.0480 [0.0391; 0.0583]
Bhaya, 2010	54	1059	0.0510 [0.0385; 0.0660]
**Total (95% CI)**		**48939**	**0.0206 [0.0133; 0.0319]**
Heterogeneity: $Tau^2 = 0.5565$; $Chi^2 = 425.23$, df = 10 ($P < 0.01$); $I^2 = 98\%$			
**European**			
Atalay, 2019	59	2550	0.0231 [0.0177; 0.0297]
**Total (95% CI)**		**214913**	**0.0242 [0.0195; 0.0299]**
Heterogeneity: $Tau^2 = 0.7080$; $Chi^2 = 2918.29$, df = 58 ($P = 0$); $I^2 = 98\%$			
Test for subgroup differences: $Chi^2 = 7.81$, df = 5 ($P = 0.17$)			

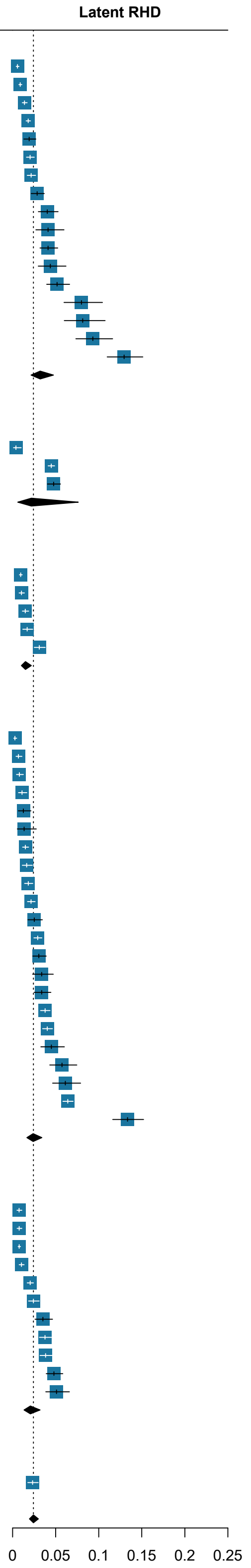

S. Figure 4: Prevalence by continents

Study or Subgroup	Events	Total	Proportion [95% CI]
**Africa**			
Ekure, 2019	11	4107	0.0027 [0.0013; 0.0048]
Mucumbitsi, 2017	17	2501	0.0068 [0.0040; 0.0109]
Marijon, 2009	17	2170	0.0078 [0.0046; 0.0125]
Ali, 2022	47	4572	0.0103 [0.0076; 0.0136]
Kane, 2012	22	2004	0.0110 [0.0069; 0.0166]
Musuku, 2018	13	1037	0.0125 [0.0067; 0.0213]
Campanale, 2017	7	522	0.0134 [0.0054; 0.0274]
Ali, 2018	66	4498	0.0147 [0.0114; 0.0186]
Beaton, 2012	72	4869	0.0148 [0.0116; 0.0186]
Ngaïdé, 2015	33	2019	0.0163 [0.0113; 0.0229]
Ali, 2018	36	2129	0.0169 [0.0119; 0.0233]
Yadeta, 2016	59	3238	0.0182 [0.0139; 0.0234]
Chillo, 2023	95	4436	0.0214 [0.0174; 0.0261]
Scheel, 2018	37	1470	0.0252 [0.0178; 0.0345]
Hunter, 2021	153	5255	0.0291 [0.0247; 0.0340]
Engel, 2015	61	2000	0.0305 [0.0234; 0.0390]
Kotit, 2017	95	3062	0.0310 [0.0252; 0.0378]
Kazahura, 2021	32	949	0.0337 [0.0232; 0.0473]
Sanyahumbi, 2016	49	1450	0.0338 [0.0251; 0.0444]
Beaton, 2015	180	4773	0.0377 [0.0325; 0.0435]
Godown, 2015	192	4773	0.0402 [0.0348; 0.0462]
Ploutz, 2015	43	956	0.0450 [0.0327; 0.0601]
Johannsen, 2021	52	907	0.0573 [0.0431; 0.0745]
Gemechu, 2017	55	898	0.0612 [0.0465; 0.0790]
Kaltenborn, 2023	425	6631	0.0641 [0.0583; 0.0703]
Lu, 2015	192	1439	0.1334 [0.1163; 0.1521]
Condemi, 2019	137	639	0.2144 [0.1832; 0.2483]
**Total (95% CI)**		**73304**	**0.0246 [0.0173; 0.0348]**
Heterogeneity: $Tau^2 = 0.8687$; $Chi^2 = 1432.1$, df = 26 ($P < 0.01$); $I^2 = 98\%$			
**Oceania**			
Bertaina, 2017	49	8684	0.0056 [0.0042; 0.0075]
Baroux, 2013	114	12803	0.0089 [0.0074; 0.0107]
Corsenac, 2016	97	7002	0.0139 [0.0112; 0.0169]
Allen, 2017	207	11434	0.0181 [0.0157; 0.0207]
Colquhoun, 2014	32	1666	0.0192 [0.0132; 0.0270]
Roberts, 2015	80	3946	0.0203 [0.0161; 0.0252]
Engelman, 2016	57	2004	0.0284 [0.0216; 0.0367]
Mirabel, 2015	49	1217	0.0403 [0.0299; 0.0529]
Voleti, 2021	26	632	0.0411 [0.0270; 0.0597]
Chatard, 2020	63	1530	0.0412 [0.0318; 0.0524]
Cramp, 2012	30	685	0.0438 [0.0297; 0.0619]
Webb, 2011	59	1142	0.0517 [0.0396; 0.0661]
Francis, 2020	49	613	0.0799 [0.0597; 0.1043]
Francis, 2021	45	553	0.0814 [0.0600; 0.1074]
Francis, 2023	71	762	0.0932 [0.0735; 0.1161]
Huang, 2017	137	1058	0.1295 [0.1098; 0.1512]
**Total (95% CI)**		**55731**	**0.0328 [0.0215; 0.0496]**
Heterogeneity: $Tau^2 = 0.7606$; $Chi^2 = 962.75$, df = 15 ($P < 0.01$); $I^2 = 98\%$			
**Latin America**			
Splitzer, 2015	4	1023	0.0039 [0.0011; 0.0100]
Nascimento, 2018	541	12048	0.0449 [0.0413; 0.0488]
Paar, 2010	150	3150	0.0476 [0.0404; 0.0556]
**Total (95% CI)**		**16221**	**0.0218 [0.0060; 0.0763]**
Heterogeneity: $Tau^2 = 1.2627$; $Chi^2 = 25.1$, df = 2 ($P < 0.01$); $I^2 = 92\%$			
**Asia**			
Karki, 2021	30	3973	0.0076 [0.0051; 0.0108]
Kumari, 2013	32	4213	0.0076 [0.0052; 0.0107]
Saxena, 2017	125	16294	0.0077 [0.0064; 0.0091]
Hosseini, 2022	143	15130	0.0095 [0.0080; 0.0111]
Shrestha, 2016	53	5178	0.0102 [0.0077; 0.0134]
Saxena, 2011	128	6270	0.0204 [0.0171; 0.0242]
Marijon, 2007	79	3677	0.0215 [0.0170; 0.0267]
Atalay, 2019	59	2550	0.0231 [0.0177; 0.0297]
Francis, 2023	62	2567	0.0242 [0.0186; 0.0309]
Davis, 2018	48	1365	0.0352 [0.0260; 0.0464]
Bhaya, 2019	113	3000	0.0377 [0.0311; 0.0451]
Choudhary, 2021	115	3000	0.0383 [0.0317; 0.0458]
Francis, 2021	97	2020	0.0480 [0.0391; 0.0583]
Bhaya, 2010	54	1059	0.0510 [0.0385; 0.0660]
**Total (95% CI)**		**70296**	**0.0197 [0.0137; 0.0282]**
Heterogeneity: $Tau^2 = 0.4781$; $Chi^2 = 521.35$, df = 13 ($P < 0.01$); $I^2 = 98\%$			
**Total (95% CI)**		**215552**	**0.0251 [0.0201; 0.0314]**
Heterogeneity: $Tau^2 = 0.7944$; $Chi^2 = 3397.16$, df = 59 ($P = 0$); $I^2 = 98\%$			
Test for subgroup differences: $Chi^2 = 3.29$, df = 3 ($P = 0.35$)			

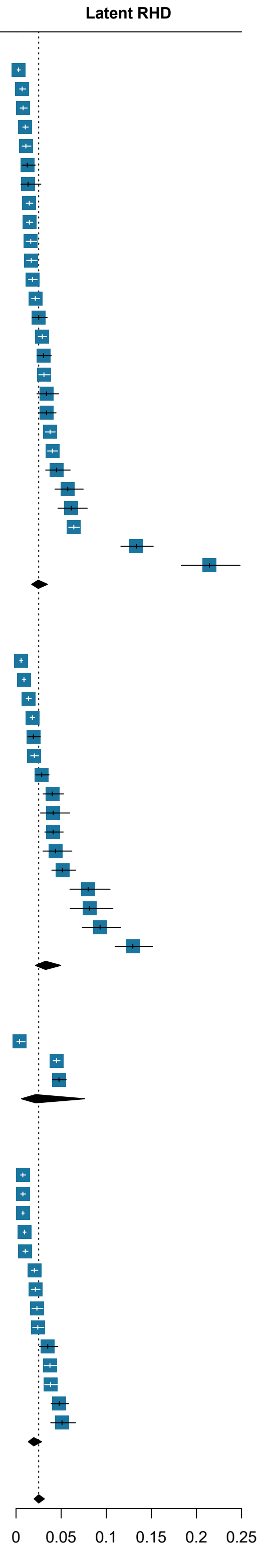

S. Figure 5: Comparison between boys and girls for the definite RHD diagnosis

Study	Boys Events	Boys Total	Girls Events	Girls Total	Weight	RR [95% CI]
Davis, 2018	5	640	20	725	3.7%	0.28 [0.11; 0.75]
Colquhoun, 2014	4	841	10	825	2.6%	0.39 [0.12; 1.25]
Engelman, 2016	4	973	10	1031	2.6%	0.42 [0.13; 1.35]
Francis, 2023	15	1569	32	1760	9.5%	0.53 [0.29; 0.97]
Atalay, 2019	13	1211	26	1339	8.1%	0.55 [0.29; 1.07]
Paar, 2010	2	1619	3	1531	1.1%	0.63 [0.11; 3.77]
Francis, 2020	13	315	19	298	7.5%	0.65 [0.33; 1.29]
Yadeta, 2016	18	1669	26	1569	9.9%	0.65 [0.36; 1.18]
Allen, 2017	49	5591	66	5843	26.0%	0.78 [0.54; 1.12]
Corsenac, 2016	26	3564	27	3341	12.2%	0.90 [0.53; 1.54]
Ngaïdé, 2015	6	1203	4	806	2.2%	1.00 [0.28; 3.55]
Beaton, 2015	22	566	25	668	11.2%	1.04 [0.59; 1.82]
Sanyahumbi, 2016	5	684	5	766	2.3%	1.12 [0.33; 3.85]
Mucumbitsi, 2017	3	1225	1	1276	0.7%	3.12 [0.33; 30.00]
Ekure, 2019	2	1901	0	2206	0.4%	5.80 [0.28; 120.78]
**Total (95% CI)**		**23571**		**23984**	**100.0%**	**0.71 [0.59; 0.86]**

Heterogeneity: $Tau^2 < 0.0001$; $Chi^2 = 13.90$, df = 14 ($P = 0.46$); $I^2 = 0\%$

Test for overall effect: $Z = -3.55$ ($P < 0.01$)

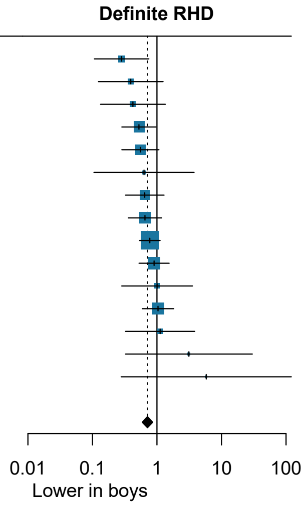

S. Figure 6: Prevalence of latent RHD by age: 10 and older X 10 and younger

Study or Subgroup	Events	Total	Proportion [95% CI]
**10 and older**			
Ekure, 2019	8	2726	0.003 [0.001; 0.006]
Mucumbitsi, 2017	12	1573	0.008 [0.004; 0.013]
Kumari, 2013	30	3147	0.010 [0.006; 0.014]
Kane, 2012	9	888	0.010 [0.005; 0.019]
Ali, 2022	47	4572	0.010 [0.008; 0.014]
Baroux, 2013	3	267	0.011 [0.002; 0.032]
Yadeta, 2016	40	2759	0.014 [0.010; 0.020]
Ali, 2018	66	4498	0.015 [0.011; 0.019]
Ali, 2018	36	2129	0.017 [0.012; 0.023]
Chillo, 2023	66	2513	0.026 [0.020; 0.033]
Hunter, 2021	153	5255	0.029 [0.025; 0.034]
Davis, 2018	29	952	0.030 [0.020; 0.043]
Engelman, 2016	36	1046	0.034 [0.024; 0.047]
Sanyahumbi, 2016	28	772	0.036 [0.024; 0.052]
Roberts, 2015	60	1629	0.037 [0.028; 0.047]
Bhaya, 2019	113	3000	0.038 [0.031; 0.045]
Scheel, 2018	31	814	0.038 [0.026; 0.054]
Choudhary, 2021	115	3000	0.038 [0.032; 0.046]
Chatard, 2020	63	1530	0.041 [0.032; 0.052]
Francis, 2023	101	2220	0.045 [0.037; 0.055]
Webb, 2011	59	1142	0.052 [0.040; 0.066]
Francis, 2020	28	367	0.076 [0.051; 0.108]
Condemi, 2019	137	639	0.214 [0.183; 0.248]
Ngaïdé, 2015	.	806	
**Total (95% CI)**		**48244**	**0.025 [0.017; 0.036]**
Heterogeneity: $Tau^2 = 0.7937$; $Chi^2 = 813.28$, df = 22 ($P < 0.01$); $I^2 = 97\%$			
**10 and younger**			
Ekure, 2019	3	1381	0.002 [0.000; 0.006]
Kumari, 2013	5	1066	0.005 [0.002; 0.011]
Mucumbitsi, 2017	5	928	0.005 [0.002; 0.013]
Bertaina, 2017	49	8684	0.006 [0.004; 0.007]
Yadeta, 2016	4	476	0.008 [0.002; 0.021]
Baroux, 2013	109	12231	0.009 [0.007; 0.011]
Scheel, 2018	6	656	0.009 [0.003; 0.020]
Karki, 2021	22	2111	0.010 [0.007; 0.016]
Corsenac, 2016	97	7002	0.014 [0.011; 0.017]
Chillo, 2023	29	1855	0.016 [0.010; 0.022]
Francis, 2020	4	246	0.016 [0.004; 0.041]
Roberts, 2015	40	2317	0.017 [0.012; 0.023]
Davis, 2018	9	413	0.022 [0.010; 0.041]
Engelman, 2016	21	958	0.022 [0.014; 0.033]
Francis, 2023	32	1109	0.029 [0.020; 0.040]
Sanyahumbi, 2016	21	678	0.031 [0.019; 0.047]
**Total (95% CI)**		**42111**	**0.011 [0.008; 0.016]**
Heterogeneity: $Tau^2 = 0.3663$; $Chi^2 = 120.23$, df = 15 ($P < 0.01$); $I^2 = 88\%$			
**Total (95% CI)**		**90355**	**0.018 [0.014; 0.024]**
Heterogeneity: $Tau^2 = 0.7984$; $Chi^2 = 1305.01$, df = 38 ($P < 0.01$); $I^2 = 97\%$			
Test for subgroup differences: $Chi^2 = 9.73$, df = 1 ($P < 0.01$)			

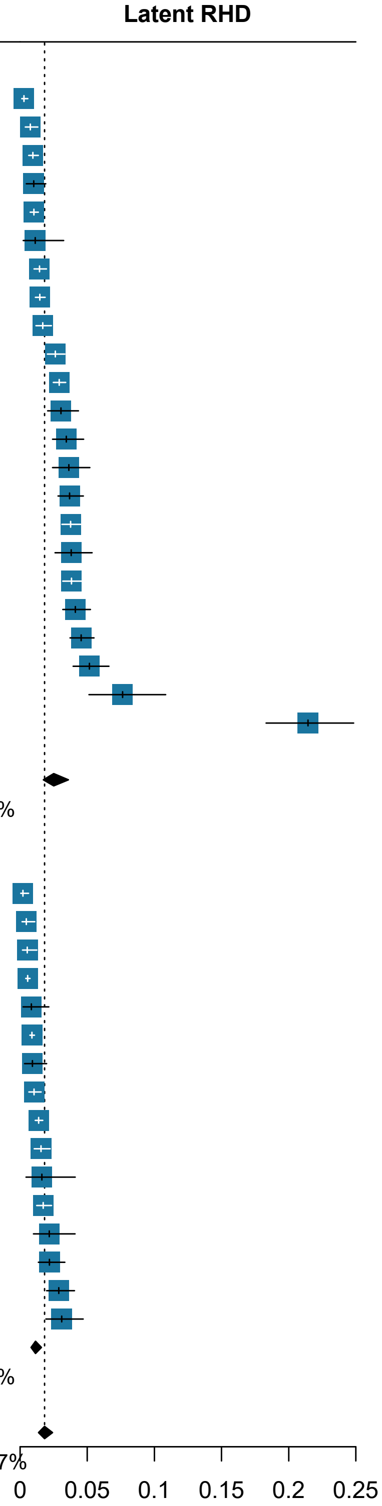

S. Figure 7: Prevalence by the WHF diagnostic criteria.[27]

Prevalence of definite RHD by different criteria from WHF	
A<br>• Pathological MR<br>• 2 morphological features of RHD	82% (CI: 75 - 87)
B<br>• MS mean gradient > 4 mmHg	1·2% (CI: 0·4 - 3·7)
C<br>• Pathological AR and<br>2 morphological features of RHD	7·2% (CI: 4·5 – 11)
D<br>• Borderline disease for both AV and MV	8·0% (CI: 4·7 – 13)

WHF criteria for definite RHD (either A, B, C or D)

A – Pathological MR + ≥ 2 morphological features of RHD of the MV
B – MS mean gradient ≥ 4 mmHg
C- Pathological AR + ≥ 2 morphological features of RHD of the AV
D- Borderline disease of both the AV and MV

WHF criteria for borderline RHD (either A, B or C)
A – Two or more morphological features of RHD of the MV
B – Pathological MR
C – Pathological AR

Features in MV

- AMVL thickening ≥ 3 mm (age specific)
- Chordal thickening
- Restrictive leaflet motion
- Excessive leaflet tip motion during systole

Features in AV

- Irregular or focal thickening
- Coaptation defect
- Restricted leaflet motion
- Prolapse

Study	Events	Total	Proportion [95% CI]
Splitzer, 2015	0	1	0.000 [0.000; 0.975]
Ploutz, 2015	5	11	0.455 [0.167; 0.766]
Chatard, 2020	22	37	0.595 [0.421; 0.752]
Allen, 2017	70	115	0.609 [0.513; 0.698]
Bhaya, 2019	60	90	0.667 [0.559; 0.763]
Choudhary, 2021	60	88	0.682 [0.574; 0.777]
Nascimento, 2018	43	63	0.683 [0.553; 0.794]
Huang, 2017	28	41	0.683 [0.519; 0.819]
Beaton, 2015	34	47	0.723 [0.574; 0.844]
Campanale, 2017	3	4	0.750 [0.194; 0.994]
Mucumbitsi, 2017	3	4	0.750 [0.194; 0.994]
Roberts, 2015	26	34	0.765 [0.588; 0.893]
Atalay, 2019	30	39	0.769 [0.607; 0.889]
Engelman, 2016	11	14	0.786 [0.492; 0.953]
Francis, 2023	37	47	0.787 [0.643; 0.893]
Hunter, 2021	39	49	0.796 [0.657; 0.898]
Ngaïdé, 2015	8	10	0.800 [0.444; 0.975]
Ali, 2018	18	22	0.818 [0.597; 0.948]
Ekure, 2019	2	2	1.000 [0.158; 1.000]
Musuku, 2018	3	3	1.000 [0.292; 1.000]
Yadeta, 2016	39	44	0.886 [0.754; 0.962]
Ali, 2018	4	4	1.000 [0.398; 1.000]
Francis, 2021	74	82	0.902 [0.817; 0.957]
Kotit, 2017	57	60	0.950 [0.861; 0.990]
Voleti, 2021	9	9	1.000 [0.664; 1.000]
Sanyahumbi, 2016	10	10	1.000 [0.692; 1.000]
Condemi, 2019	17	17	1.000 [0.805; 1.000]
Shrestha, 2016	35	36	0.972 [0.855; 0.999]
Remenyi, 2019	25	25	1.000 [0.863; 1.000]
Francis, 2020	.	32	
Kazahura, 2021	.	18	
Godown, 2015	.	52	
**Total (95% CI)**		**1110**	**0.822 [0.754; 0.874]**

Heterogeneity: $Tau^2 = 0.7116$; $Chi^2 = 56.01$, df = 28 (P < 0.01); $I^2 = 50\%$

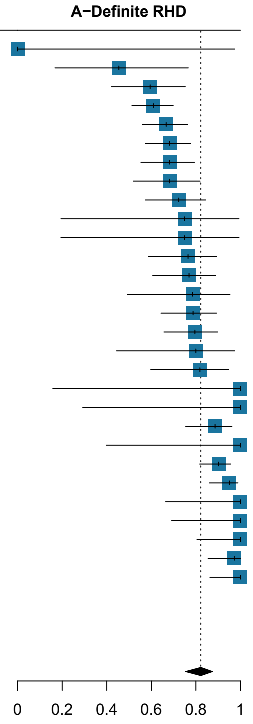

Study	Events	Total	Proportion [95% CI]
Bhaya, 2019	0	90	0.000 [0.000; 0.040]
Choudhary, 2021	0	88	0.000 [0.000; 0.041]
Nascimento, 2018	0	63	0.000 [0.000; 0.057]
Kotit, 2017	0	60	0.000 [0.000; 0.060]
Hunter, 2021	0	49	0.000 [0.000; 0.073]
Francis, 2021	1	82	0.012 [0.000; 0.066]
Chatard, 2020	0	37	0.000 [0.000; 0.095]
Shrestha, 2016	0	36	0.000 [0.000; 0.097]
Remenyi, 2019	0	25	0.000 [0.000; 0.137]
Ali, 2018	0	22	0.000 [0.000; 0.154]
Atalay, 2019	1	39	0.026 [0.001; 0.135]
Condemi, 2019	0	17	0.000 [0.000; 0.195]
Ploutz, 2015	0	11	0.000 [0.000; 0.285]
Ngaïdé, 2015	0	10	0.000 [0.000; 0.308]
Sanyahumbi, 2016	0	10	0.000 [0.000; 0.308]
Voleti, 2021	0	9	0.000 [0.000; 0.336]
Yadeta, 2016	3	44	0.068 [0.014; 0.187]
Engelman, 2016	1	14	0.071 [0.002; 0.339]
Huang, 2017	3	41	0.073 [0.015; 0.199]
Allen, 2017	9	115	0.078 [0.036; 0.143]
Beaton, 2015	4	47	0.085 [0.024; 0.204]
Roberts, 2015	3	34	0.088 [0.019; 0.237]
Ali, 2018	0	4	0.000 [0.000; 0.602]
Campanale, 2017	0	4	0.000 [0.000; 0.602]
Mucumbitsi, 2017	0	4	0.000 [0.000; 0.602]
Musuku, 2018	0	3	0.000 [0.000; 0.708]
Francis, 2023	6	47	0.128 [0.048; 0.257]
Ekure, 2019	0	2	0.000 [0.000; 0.842]
Splitzer, 2015	0	1	0.000 [0.000; 0.975]
Francis, 2020	.	32	
Kazahura, 2021	.	18	
Godown, 2015	.	52	
**Total (95% CI)**		**1110**	**0.012 [0.004; 0.037]**

Heterogeneity: $Tau^2 = 2.0655$; $Chi^2 = 6.72$, df = 28 (P = 1.00); $I^2 = 0\%$

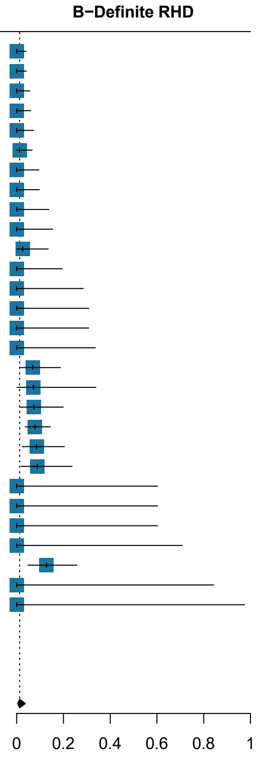

Study	Events	Total	Proportion [95% CI]
Chatard, 2020	0	37	0.000 [0.000; 0.095]
Remenyi, 2019	0	25	0.000 [0.000; 0.137]
Beaton, 2015	1	47	0.021 [0.001; 0.113]
Condemi, 2019	0	17	0.000 [0.000; 0.195]
Shrestha, 2016	1	36	0.028 [0.001; 0.145]
Kotit, 2017	2	60	0.033 [0.004; 0.115]
Francis, 2021	3	82	0.037 [0.008; 0.103]
Ploutz, 2015	0	11	0.000 [0.000; 0.285]
Ali, 2018	1	22	0.045 [0.001; 0.228]
Sanyahumbi, 2016	0	10	0.000 [0.000; 0.308]
Voleti, 2021	0	9	0.000 [0.000; 0.336]
Huang, 2017	3	41	0.073 [0.015; 0.199]
Nascimento, 2018	5	63	0.079 [0.026; 0.176]
Roberts, 2015	3	34	0.088 [0.019; 0.237]
Ali, 2018	0	4	0.000 [0.000; 0.602]
Campanale, 2017	0	4	0.000 [0.000; 0.602]
Ngaïdé, 2015	1	10	0.100 [0.003; 0.445]
Francis, 2023	5	47	0.106 [0.035; 0.231]
Musuku, 2018	0	3	0.000 [0.000; 0.708]
Yadeta, 2016	6	44	0.136 [0.052; 0.274]
Engelman, 2016	2	14	0.143 [0.018; 0.428]
Hunter, 2021	7	49	0.143 [0.059; 0.272]
Atalay, 2019	6	39	0.154 [0.059; 0.305]
Allen, 2017	19	115	0.165 [0.103; 0.246]
Ekure, 2019	0	2	0.000 [0.000; 0.842]
Mucumbitsi, 2017	1	4	0.250 [0.006; 0.806]
Bhaya, 2019	25	90	0.278 [0.189; 0.382]
Choudhary, 2021	25	88	0.284 [0.193; 0.390]
Splitzer, 2015	1	1	1.000 [0.025; 1.000]
Francis, 2020	.	32	
Kazahura, 2021	.	18	
Godown, 2015	.	52	
**Total (95% CI)**		**1110**	**0.072 [0.045; 0.113]**

Heterogeneity: $Tau^2 = 0.7621$; $Chi^2 = 49.80$, df = 28 ($P < 0.01$); $I^2 = 44\%$

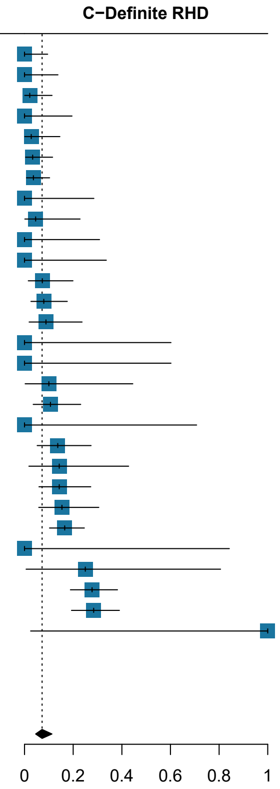

Study	Events	Total	Proportion [95% CI]
Shrestha, 2016	0	36	0.000 [0.000; 0.097]
Kotit, 2017	1	60	0.017 [0.000; 0.089]
Condemi, 2019	0	17	0.000 [0.000; 0.195]
Engelman, 2016	0	14	0.000 [0.000; 0.232]
Choudhary, 2021	3	88	0.034 [0.007; 0.096]
Sanyahumbi, 2016	0	10	0.000 [0.000; 0.308]
Francis, 2021	4	82	0.049 [0.013; 0.120]
Voleti, 2021	0	9	0.000 [0.000; 0.336]
Atalay, 2019	2	39	0.051 [0.006; 0.173]
Bhaya, 2019	5	90	0.056 [0.018; 0.125]
Roberts, 2015	2	34	0.059 [0.007; 0.197]
Hunter, 2021	3	49	0.061 [0.013; 0.169]
Ali, 2018	0	4	0.000 [0.000; 0.602]
Mucumbitsi, 2017	0	4	0.000 [0.000; 0.602]
Ngaïdé, 2015	1	10	0.100 [0.003; 0.445]
Remenyi, 2019	3	25	0.120 [0.025; 0.312]
Musuku, 2018	0	3	0.000 [0.000; 0.708]
Ali, 2018	3	22	0.136 [0.029; 0.349]
Allen, 2017	17	115	0.148 [0.089; 0.226]
Ekure, 2019	0	2	0.000 [0.000; 0.842]
Beaton, 2015	8	47	0.170 [0.076; 0.308]
Huang, 2017	7	41	0.171 [0.072; 0.321]
Nascimento, 2018	15	63	0.238 [0.140; 0.362]
Campanale, 2017	1	4	0.250 [0.006; 0.806]
Splitzer, 2015	0	1	0.000 [0.000; 0.975]
Francis, 2023	12	47	0.255 [0.139; 0.403]
Chatard, 2020	15	37	0.405 [0.248; 0.579]
Ploutz, 2015	6	11	0.545 [0.234; 0.833]
Francis, 2020	.	32	
Kazahura, 2021	.	18	
Yadeta, 2016	.	44	
Godown, 2015	.	52	
**Total (95% CI)**		**1110**	**0.080 [0.047; 0.131]**

Heterogeneity: $Tau^2 = 1.1342$; $Chi^2 = 66.35$, df = 27 ($P < 0.01$); $I^2 = 59\%$

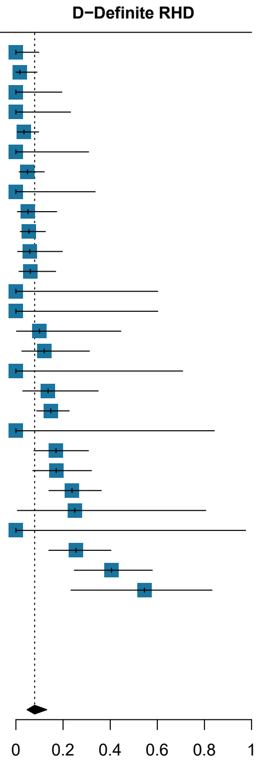

S. Figure 8: Leave-one-out of the comparison between subgroups

A) Boys *vs*. Girls

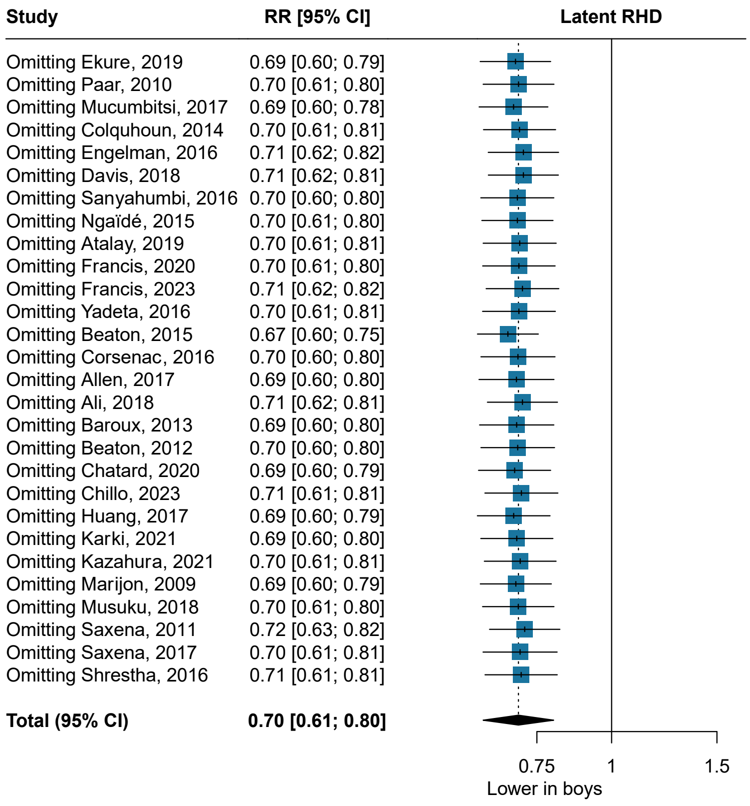

B) Urban *vs*. Rural

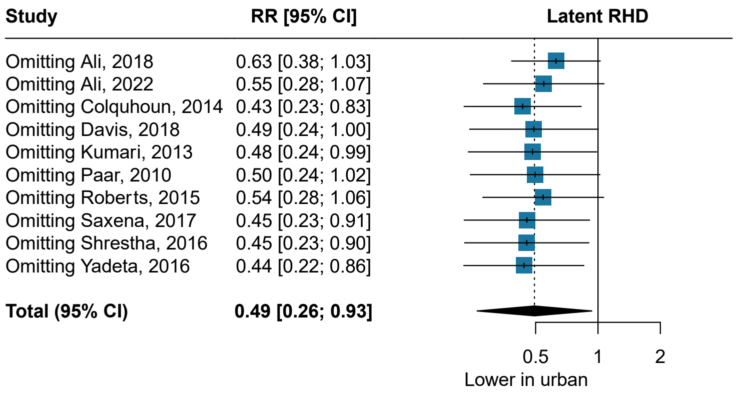

C) Public *vs*. Private

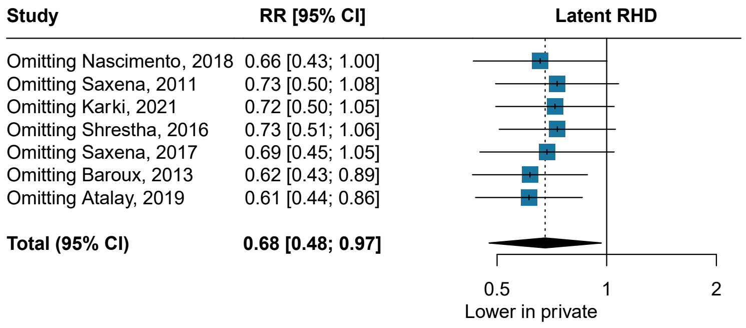

D) Low-income *vs*. High-income

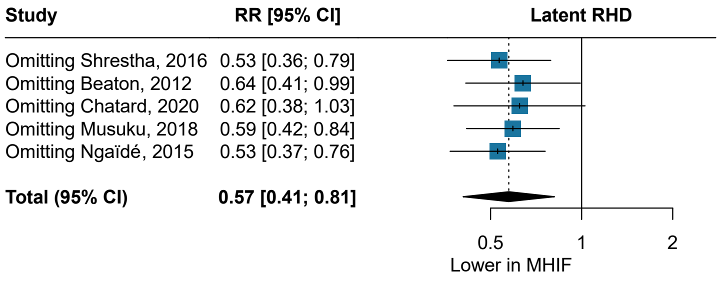

S. Figure 9: Funnel plot of the primary outcome

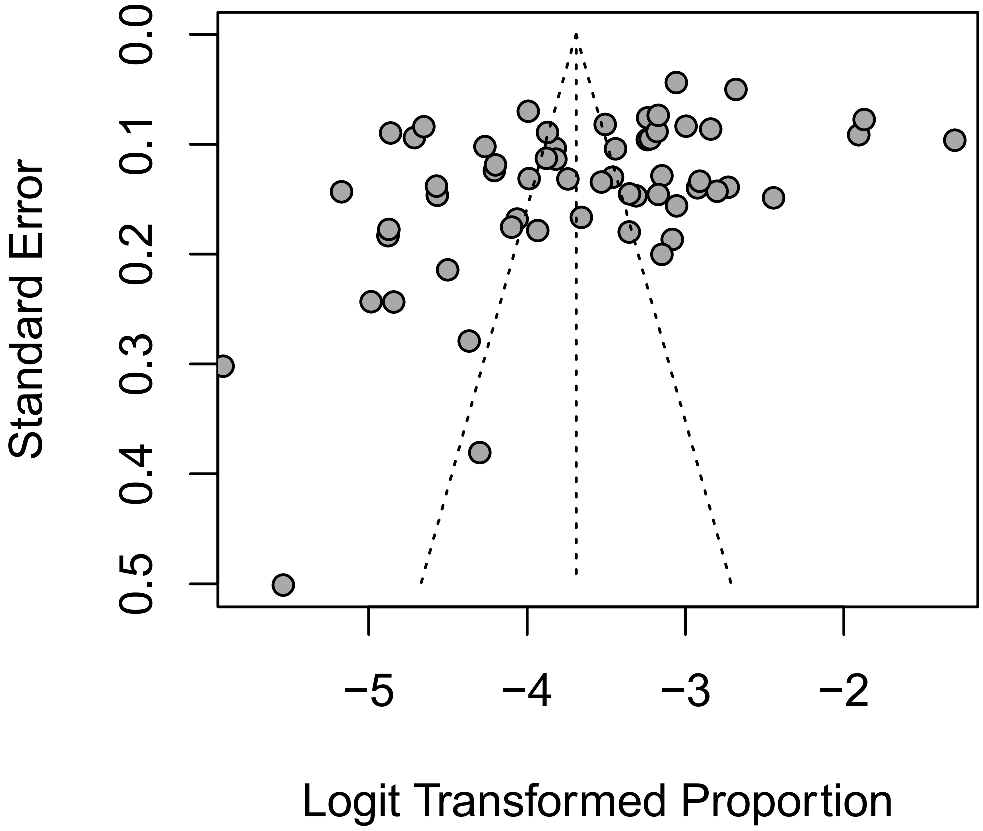

References:

1. Ahmed J, Mostafa Zaman M, Monzur Hassan MM. Prevalence of rheumatic fever and rheumatic heart disease in rural Bangladesh. Trop Doct. 2005 Jul;35(3):160–1.

2. Anabwani GM, Bonhoeffer P. Prevalence of heart disease in school children in rural Kenya using colour-flow echocardiography. East Afr Med J. 1996 Apr;73(4):215–7.

3. Fakakovikaetau T, Latu ST, Gentles T, Wilson N, Hope S, Cox S, et al. PM014 Echocardiography Screening For RHD of 5 – 15 Years Old Children Saves Lives. Global Heart. 2016 Jun 1;11(2, Supplement):e71.

4. Farrag AAM, El Aroussy WA, El Hagracy N, Fawzy H, Taha N. PM472 Echocardiographic screening for Rheumatic Valvular Heart Disease among Egyptian School Children. Global Heart. 2014 Mar 1;9(1, Supplement):e158–9.
5. Kwok M, Chow C, Henriksen K, Foster S, Jones A, Bentley-Taylor M, et al. PREVALENCE OF RHEUMATIC HEART DISEASE IN URBAN AND RURAL ANGOLA BY ECHOCARDIOGRAPHY. Canadian Journal of Cardiology. 2014 Oct 1;30(10):S104–5.
6. Rossi E, Felici AR, Banteyrga L. Subclinical rheumatic heart disease in an Eritrean high-school population, detected by echocardiography. J Heart Valve Dis. 2014 Mar;23(2):235–9.
7. Viali S, Futi V, Lafaele T. PM293 Prevalence of Rheumatic Heart Disease Among Secondary School Children in Samoa Detected by Echocardiography Screening. Global Heart. 2016 Jun 1;11(2, Supplement):e120.
8. Sunday 28 August 2016. European Heart Journal. 2016 Aug 1;37(suppl_1):191–598.
9. Min S, Carrillo B, Lopez K, Francisco Guido R, yeung michael, Espinoza C, et al. Abstract 14599: A Pilot Rheumatic Heart Disease Screening Program Using Portable, Handheld Echocardiography in Rivas, Nicaragua. Circulation. 2023 Nov 7;148(Suppl_1):A14599–A14599.
10. Sriharibabu M, Himabindu Y, Lakshmanrao N. Prevalence and Spectrum of Cardiac Disease in a Rural Populace of Coastal Andhra Pradesh. Indian Journal of Public Health Research & Development. 2014 Apr 1;5:198.
11. Zhimin W, Yubao Z, Lei S, Xianliang Z, Wei Z, Li S, et al. Prevalence of chronic rheumatic heart disease in Chinese adults. Int J Cardiol. 2006 Mar 8;107(3):356–9.
12. Longo-Mbenza B, Bayekula M, Ngiyulu R, Kintoki VE, Bikangi NF, Seghers KV, et al. Survey of rheumatic heart disease in school children of Kinshasa town. International Journal of Cardiology. 1998 Feb 28;63(3):287–94.
13. Zaman MM, Choudhury SR, Rahman S, Ahmed J. Prevalence of rheumatic fever and rheumatic heart disease in Bangladeshi children. Indian Heart J. 2015;67(1):45–9.
14. Amade ES, Lichucha E, Ossman Z, Jamal K, Mastala A, Thorup L, et al. Leveraging School Health Programs in Africa: Integrated Screening for Rheumatic Heart Disease and Dental Caries. Ann of Global Health [Internet]. 2023;89(1). Available from: https://www.embase.com/search/results?subaction=viewrecord&id=L2026745357&from=export
15. Schaffer WL, Galloway JM, Roman MJ, Palmieri V, Liu JE, Lee ET, et al. Prevalence and correlates of rheumatic heart disease in American Indians (the Strong Heart Study). Am J Cardiol. 2003 Jun 1;91(11):1379–82.
16. Zühlke L, Karthikeyan G, Engel ME, Rangarajan S, Mackie P, Cupido-Katya Mauff B, et al. Clinical Outcomes in 3343 Children and Adults With Rheumatic Heart Disease From 14 Low- and Middle-Income Countries: Two-Year Follow-Up of the Global Rheumatic Heart Disease Registry (the REMEDY Study). Circulation. 2016 Nov 8;134(19):1456–66.
17. Sanyahumbi A, Beaton A, Guffey D, Hosseinipour MC, Karlsten M, Minard CG, et al. Two-year evolution of latent rheumatic heart disease in Malawi. Congenit Heart Dis. 2019 Jul;14(4):614–8.
18. Hempenstall A, Howell E, Kang K, Chau KWT, Browne A, Kris E, et al. Echocardiographic Screening Detects a Significant Burden of Rheumatic Heart Disease

in Australian Torres Strait Islander Children and Missed Opportunities for its Prevention. Am J Trop Med Hyg. 2021 Jan 11;104(4):1211–4.
19. Laudari S, Tiwari KK, Pazdernik M, Sharma SK. Rheumatic Heart Disease Screening Among School Children in Central Nepal. JACC Case Rep. 2019 Aug 21;1(2):218–20.
20. Mirabel M, Fauchier T, Bacquelin R, Tafflet M, Germain A, Robillard C, et al. Echocardiography screening to detect rheumatic heart disease: A cohort study of schoolchildren in French Pacific Islands. International Journal of Cardiology. 2015 Jun 1;188:89–95.
21. Shrestha NR, Uranw S, Karki P, Bastola S, Mahato R, Sherpa K, et al. Prevalence of latent structural heart disease in Nepali schoolchildren. Cardiology in the Young. 2022 Jul;32(7):1151–3.
22. Zühlke L, Engel ME, Lemmer CE, van de Wall M, Nkepu S, Meiring A, et al. The natural history of latent rheumatic heart disease in a 5 year follow-up study: a prospective observational study. BMC Cardiovasc Disord. 2016 Feb 19;16:46.
23. Remenyi B, Davis K, Draper A, Bayley N, Paratz E, Reeves B, et al. Single Parasternal-Long-Axis-View-Sweep Screening Echocardiographic Protocol to Detect Rheumatic Heart Disease: A Prospective Study of Diagnostic Accuracy. Heart Lung Circ. 2020 Jun;29(6):859–66.
24. Roberts K, Maguire G, Brown A, Atkinson D, Reményi B, Wheaton G, et al. Echocardiographic screening for rheumatic heart disease in high and low risk Australian children. Circulation. 2014 May 13;129(19):1953–61.
25. Miranda LP, Camargos PAM, Torres RM, Meira ZMA. Prevalence of Rheumatic Heart Disease in a Public School of Belo Horizonte. Arq Bras Cardiol. 2014 Aug;103:89–97.
26. Reeves BM, Kado J, Brook M. High prevalence of rheumatic heart disease in Fiji detected by echocardiography screening. J Paediatr Child Health. 2011 Jul;47(7):473–8.
27. Reményi B, Wilson N, Steer A, Ferreira B, Kado J, Kumar K, et al. World Heart Federation criteria for echocardiographic diagnosis of rheumatic heart disease—an evidence-based guideline. Nat Rev Cardiol. 2012 Feb 28;9(5):297–309.
